# p97/VCP inhibition causes excessive MRE11-dependent DNA end resection promoting cell killing after ionizing radiation

**DOI:** 10.1016/j.celrep.2021.109153

**Published:** 2021-05-25

**Authors:** Susan Kilgas, Abhay Narayan Singh, Salome Paillas, Chee-Kin Then, Ignacio Torrecilla, Judith Nicholson, Lisa Browning, Iolanda Vendrell, Rebecca Konietzny, Benedikt M. Kessler, Anne E. Kiltie, Kristijan Ramadan

**Affiliations:** 1 MRC Oxford Institute for Radiation Oncology, Department of Oncology, University of Oxford, Oxford OX3 7DQ, UK; 2 Department of Cellular Pathology, Oxford University Hospitals, NHS Foundation Trust, Oxford OX3 9DU, UK; 3 TDI Mass Spectrometry Laboratory, Target Discovery Institute, Nuffield Department of Medicine, University of Oxford, Oxford OX3 7FZ, UK

**Keywords:** p97, ionizing radiation, IR, CB-5083, MRE11, DNA damage, DNA double-strand break repair, homologous recombination, single-strand annealing, bladder cancer

## Abstract

The ATPase p97 is a central component of the ubiquitin-proteasome degradation system. p97 uses its ATPase activity and co-factors to extract ubiquitinated substrates from different cellular locations, including DNA lesions, thereby regulating DNA repair pathway choice. Here, we find that p97 physically and functionally interacts with the MRE11-RAD50-NBS1 (MRN) complex on chromatin and that inactivation of p97 blocks the disassembly of the MRN complex from the sites of DNA damage upon ionizing radiation (IR). The inhibition of p97 function results in excessive 5′-DNA end resection mediated by MRE11 that leads to defective DNA repair and radiosensitivity. In addition, p97 inhibition by the specific small-molecule inhibitor CB-5083 increases tumor cell killing following IR both *in vitro* and *in vivo*. Mechanistically, this is mediated via increased MRE11 nuclease accumulation. This suggests that p97 inhibitors might be exploited to improve outcomes for radiotherapy patients.

## Highlights

• p97 interacts with MRE11 and regulates its retention on chromatin after IR
• p97 inactivation causes excessive MRE11-mediated DNA end resection
• p97-mediated MRE11 chromatin retention causes HR defects but efficient SSA
• CB-5083 radiosensitizes cells and suppresses tumor growth *in vivo* after IR

## Graphical abstract

**Figure:**
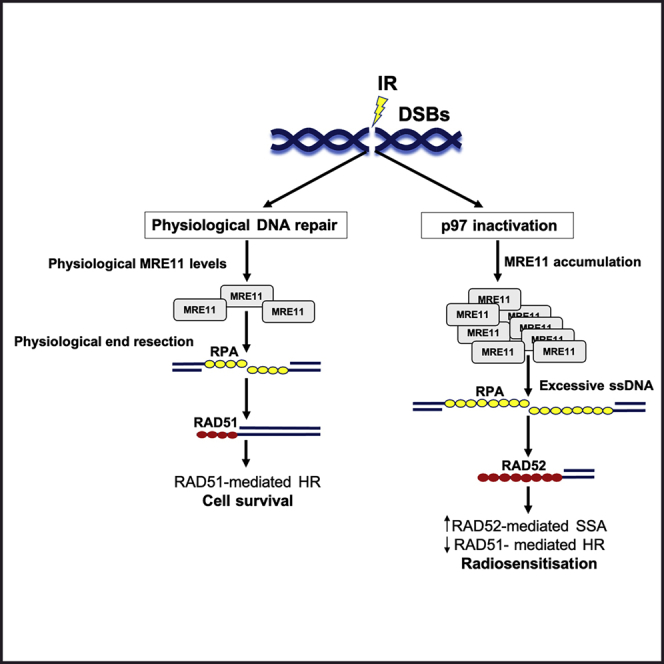

## Teaser

Kilgas et al. show that p97/VCP interacts with the MRN complex. Inactivating p97 blocks MRN disassembly from chromatin, causing MRE11 chromatin retention and excessive 5′-DNA end resection, along with HR defects and RAD52-mediated SSA. This change in DSB repair pathway choice radiosensitizes cells, which is rescued by inactivation of MRE11.

## Introduction

Cancer cells are under continuous proteotoxic stress from aggregated and misfolded proteins due to an increased number of chromosomes and point mutations in protein-coding sequences. This makes tumors strongly dependent on protein degradation pathways for survival (Guang et al., 2019; Deshaies, 2014). The ubiquitin-proteasome system (UPS) is responsible for most of the protein degradation in mammalian cells and represents over 19% of mutated cancer driver genes (Collins and Goldberg, 2017; Tokheim et al., 2021). Dysregulation of the UPS is involved in nearly all cancer hallmarks (Hanahan and Weinberg, 2011). The AAA+ ATPase p97, also known as valosin-containing protein (VCP), is the central component of the UPS. Its overexpression has been demonstrated in various tumors (Tsujimoto et al., 2004; Yamamoto et al., 2004a, 2004b, 2004c), implying that both the UPS and p97 are necessary for cancer cell survival through the maintenance of protein homeostasis.

p97, with the aid of a plethora of co-factors that contain specialized domains to bind ubiquitinated proteins, extracts ubiquitinated substrates from different cellular locations. Such extracted ubiquitinated proteins are prepared for degradation or recycled (van den Boom and Meyer, 2018; Dantuma and Hoppe, 2012; Stolz et al., 2011). Previous work on the role of p97 in cancer has largely focused on its cytoplasmic functions, mainly the endoplasmic-reticulum-associated degradation pathway (Anderson et al., 2015; Le Moigne et al., 2017; Stolz et al., 2011). However, accumulating evidence has demonstrated a role for p97 in disassembly/removal of ubiquitinated proteins from chromatin (Moreno et al., 2014; Franz et al., 2016; Cao et al., 2003; Ramadan et al., 2007; Hirsch et al., 2009). Similarly, p97 regulates DNA double-strand break (DSB) repair by affecting repair pathway choice (Kuo et al., 2016; van den Boom et al., 2016; Meerang et al., 2011; Acs et al., 2011). p97 has a function in both main DSB pathways, homologous recombination (HR) and non-homologous end joining (NHEJ). For example, p97 removes ubiquitinated KU80 from broken DNA ends, thus promoting HR over NHEJ (van den Boom et al., 2016). Conversely, p97 removes the main DNA damage response (DDR) ubiquitin ligase RNF8 from DSB sites, thereby promoting NHEJ (Singh et al., 2019).

The MRE11-RAD50-NBS1 (MRN) complex has a critical role in the initial processing of DSB ends for efficient HR repair. The endonuclease and 3′-5′ exonuclease activities of MRE11 resect double-stranded DNA (dsDNA) and create 3′ single-stranded DNA (ssDNA), an essential DNA structure for HR repair, in S/G2 phase of the cell cycle (Paull, 2018; Nieminuszczy et al., 2019). Resected DNA ends can also be processed by alternative pathways that are inherently mutagenic, namely, alternative NHEJ (alt-NHEJ) and single-strand annealing (SSA) (Ceccaldi et al., 2016; Bhargava et al., 2016). Various proteins, including UBQLN4, DYNLL1, and C1QBP, regulate MRE11 activity on chromatin (Bai et al., 2019; Jachimowicz et al., 2019; He et al., 2018). For example, UBQLN4 remodels the MRN complex to curtail DSB end resection by controlling MRE11’s association with chromatin and removing ubiquitinated MRE11 from damaged chromatin. One of the functions of MRN remodeling is to control MRE11 nuclease activity, because both decreased and excessive MRE11 nuclease activity compromises error-free DSB repair (Hashimoto et al., 2010; Ray Chaudhuri et al., 2016; Schlacher et al., 2011).

Here, we show that p97 interacts with and removes MRE11 from chromatin, thereby controlling MRE11-mediated 5′-DNA end resection after IR. Furthermore, inactivation of p97 ATPase activity results in an accumulation of MRE11 on chromatin after IR. This leads to an increase in ssDNA generation and a DNA repair template switch from error-free RAD51-mediated HR to RAD52-mediated SSA. We therefore postulate that p97-mediated regulation of MRE11 on chromatin is essential for DSB repair pathway choice, 53BP1 foci formation, and cell survival. Indeed, increased DNA end resection and radiosensitivity in p97-inactivated cells were alleviated by inactivating MRE11 nuclease activity. Finally, using bladder cancer as a model system, we demonstrate that inhibition of p97 by CB-5083 increases tumor cell killing after IR and suppresses xenograft tumor growth, without additional toxicity to surrounding normal tissues in the mouse model used. This suggests that the here-discovered p97-MRE11 axis could potentially be targeted clinically to achieve radiosensitization and improve tumor cure in patients, especially in those with elevated levels of p97 protein.

## Results

### p97 is required for MRE11 turnover on chromatin and DNA damage

**Figure 1:**
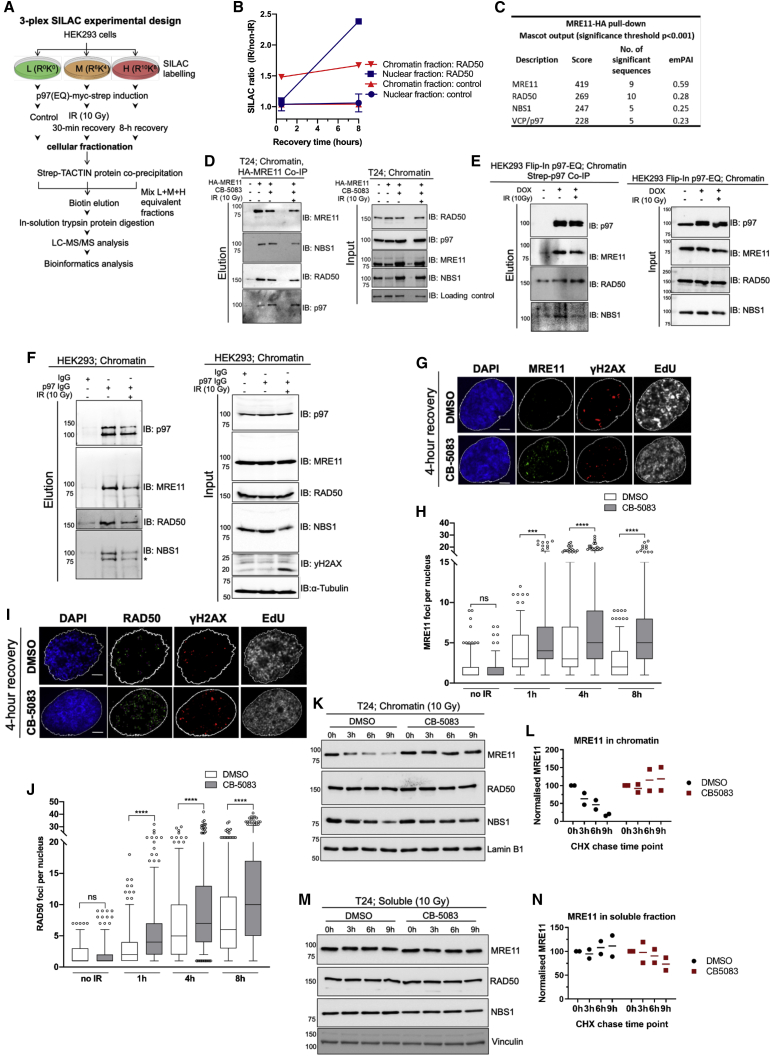
p97 is required for MRE11 turnover on chromatin and DNA damage (A) Schematic of SILAC-based mass spectrometry screen for p97 interactome after 10 Gy ionizing radiation (IR). (B) Illustrative graph based on the schematic in (A), showing SILAC ratio of p97 and RAD50 interaction on chromatin after 10 Gy IR. (C) Mass spectrometry analysis of the MRE11 interactome identifies RAD50, NBS1, and p97 peptides in similar abundance by MASCOT score. (D) p97 interacts with the MRE11-RAD50-NBS1 (MRN) complex in chromatin fraction of T24 cells. HA-tagged MRE11 was pulled down in the presence or absence of the p97 inhibitor CB-5083 (10 μM) and IR (10 Gy); n = 2. (E) Doxycycline (DOX)-inducible HEK293 Flip-In p97-EQ-Strep-tag cells were exposed to IR (10 Gy) or not and fractionated to isolate the chromatin fraction and p97-EQ-Strep; n = 2. (F) Endogenous p97 was immunoprecipitated with p97-specific immunoglobulin G (IgG) antibodies in HEK2923 cells showing p97 and MRN complex interactions on chromatin in the presence or absence of IR (10 Gy). (G) Representative images of the number of MRE11 foci at 4 h of recovery after 2 Gy IR and CB-5083 treatment (scale bars, 5 μm). (H) Quantifications of MRE11 foci in T24 cells after 2 Gy IR in the presence of p97 inhibitor (G). Data are presented as boxplot, and values beyond the 5th and 95th percentiles are shown as individual data points; n = 3 independent experiments; Mann-Whitney test. NS, not significant; ^∗∗∗^p < 0.001; ^∗∗∗∗^p < 0.0001. (I) Representative images of the number RAD50 foci at 4 h of recovery after 2 Gy IR and CB-5083 treatment (scale bars, 5 μm). (J) Quantifications of RAD50 foci as in (H). Data are presented as boxplot, and values beyond the 5th and 95th percentiles are shown as individual data points; n = 3 independent experiments; Mann-Whitney test. NS, not significant; ^∗∗∗∗^p < 0.0001. (K) Effect of CB-5083 (10 μM) on MRN complex stability on chromatin fraction after IR in CHX-exposed T24 cells; n = 2 independent experiments. (L) Quantifications of normalized MRE11 levels on chromatin. (M) Effect of CB-5083 (10 μM) on MRN complex stability in the soluble fraction (cytoplasm and nucleus pooled; after IR) in CHX-exposed T24 cells. (N) Quantifications of (M); n = 2. See also Figures S1 and S2.

p97 has many interaction partners and co-factors required for its diverse cellular functions (Schuberth and Buchberger, 2008; Yeung et al., 2008; Meyer and Weihl, 2014). However, little is known about how p97 regulates its substrates on chromatin during DNA damage repair (Meyer et al., 2012; van den Boom and Meyer, 2018; Singh et al., 2019; Meerang et al., 2011). In order to identify relevant p97 substrates on chromatin after DNA damage from IR, we designed a triplex stable isotope labeling with amino acids in cell culture (SILAC) mass spectrometry experiment (Figures 1A and 1B) using a stable transfected and doxycycline (DOX) inducible dominant-negative p97-E578Q (EQ) variant in Human Embryonic Kidney 293 (HEK293) cells. This variant is deficient in ATP hydrolysis but has the ability to bind ubiquitinated substrates, thus serving as a substrate-trapping mutant (Ye et al., 2003; Meerang et al., 2011; Ritz et al., 2011). HEK293 cells were irradiated with 10 Gy IR, recovered over 8 h, p97 was isolated and analyzed by mass spectrometry for p97 interaction partners relevant to DNA damage repair. An increased interaction between p97 and the MRN complex component RAD50 was observed on chromatin and in the nucleus during 8 h of recovery after IR (Figure 1B).

Furthermore, we performed a reciprocal mass spectrometry screen of the MRE11 interactome in T24 urinary bladder cancer cells, which identified p97 as one of the top interaction partners of MRE11, supporting our SILAC mass spectrometry findings. The number of p97 peptides in the complex with MRE11 was similar to the number of peptides of RAD50 and NBS1, the two constitutive partners of MRE11 in the MRN complex (Figure 1C; Table S1). Both mass spectrometry findings were confirmed by co-immunoprecipitation of Strep-tag p97 or hemagglutinin (HA)-MRE11 from chromatin extracts in HEK293 or T24 cells, respectively, before and after IR (Figures 1D and 1E). The physical interaction between p97 and the MRN complex was also corroborated between endogenous proteins (Figure 1F).

We hypothesized that p97 is involved in the regulation of the MRN complex on chromatin, specifically by being required for the disassembly of MRN from the chromatin. To test our hypothesis, we quantified MRE11 and RAD50 nuclear foci after IR in p97-inhibited conditions to determine whether MRN complex components accumulate on sites of DNA damage. Treatment with CB-5083, a specific small-molecule inhibitor of p97, resulted in an accumulation of both MRE11 and RAD50 (Figures 1G–1J) foci over 8 h after IR. However, the total protein levels of the MRN complex components between CB-5083- and control-treated cells remained similar (Figures S1A and S1B), suggesting that this accumulation is DNA and chromatin specific, resulting from an accumulation of the available pool of RAD50 and MRE11 that is not efficiently removed in the absence of p97.

Indeed, biochemical analysis of the MRN complex turnover by cycloheximide (CHX) chase in soluble and chromatin fractions after IR showed that chemical inhibition of p97 specifically blocks the disassembly of the MRN complex from chromatin but does not affect the overall turnover of the complex (Figures 1K–1N and S1E) in T24 cells. These results were more pronounced in small interfering RNA (siRNA) p97-depleted T24 (Figures S1C and S1D) or HeLa cells (Figure S1F), suggesting a universal mechanism of MRN chromatin disassembly by p97. In addition, the inactivation of p97 ATPase activity by a mild expression of DOX-inducible p97-EQ variant also caused accumulation of the MRN complex on chromatin, but not in the soluble pool (Figures S1G–S1I).

To further support these results, we used UV-A laser microirradiation, which creates localized DNA damage in living cells (Figure S2). Similar to our biochemical analysis, inactivation of the p97 ATPase activity caused elevated accumulation of MRE11 (Figures S2A and S2B) and RAD50 (Figures S2C and S2D) at the sites of DNA lesions. Together, these data show that p97 physically interacts with and removes the MRN complex from chromatin and sites of DNA damage.

### p97 inhibition results in excessive 5′-DNA end resection mediated by MRE11

**Figure 2:**
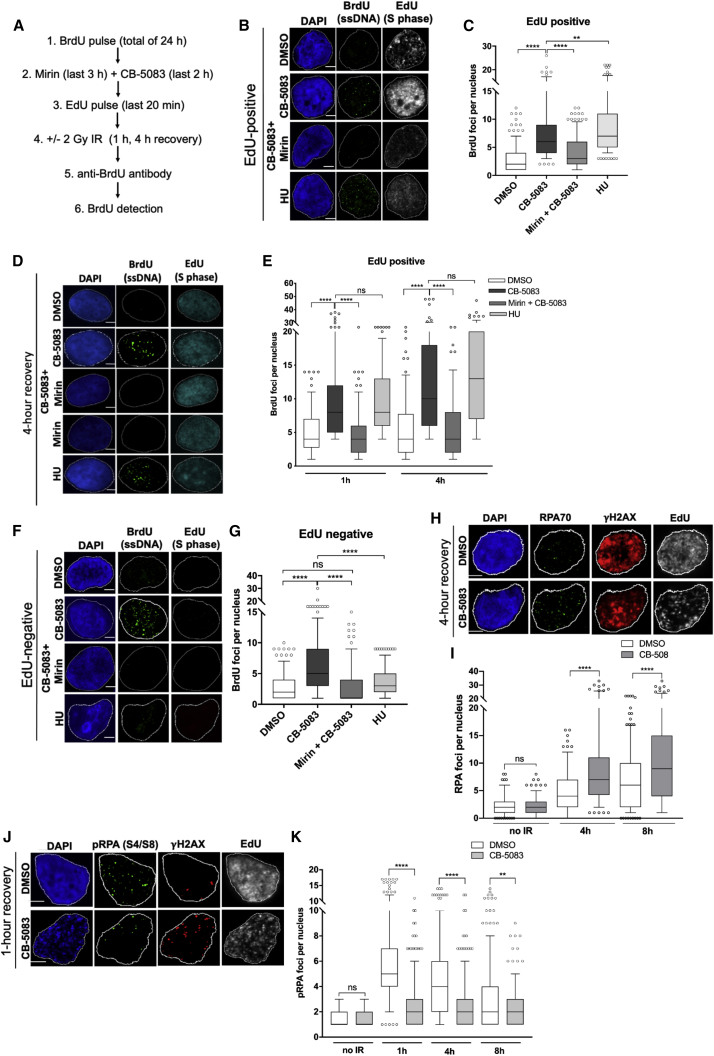
p97 inhibition results in excessive DNA end resection mediated by MRE11 (A) Schematic of the BrdU assay. (B and C) Representative images (B) and quantification of EdU-positive BrdU foci (C) in CB-5083- and mirin-treated T24 cells. Scale bars, 5 μm; n = 3. (D and E) Representative images (D) and quantification (E) of BrdU foci in EdU-positive T24 cells after 2 Gy IR and 1 or 4 h of recovery. Scale bars, 5 μm; n = 3. (F and G) Representative images (F) and quantification (G) of EdU-negative BrdU foci in CB-5083- and mirin-treated T24 cells. Scale bars, 5 μm; n = 3 (H and I) Representative images (H) and quantification (I) of EdU-positive RPA70 foci at 4 h of recovery after 2 Gy IR in CB-5083 treated T24 cells. Scale bars, 5 μm; n = 3. (J and K) Representative images (J) and quantification (K) of phosphorylated RPA foci (S4/S8) formation at 1 h of recovery from 2 Gy IR. Scale bars, 5 μm; n = 3. For all graphs, data are presented as boxplot, and values beyond the 5th and 95th percentiles are shown as individual data points. For statistical analysis in (C), (E), and (G), Kruskal-Wallis with Dunn’s multiple comparisons was used. For (I) and (K), the Mann-Whitney test was used. NS, not significant; ^∗∗^p < 0.01; ^∗∗∗∗^p < 0.0001. See also Figures S3 and S4.

We further sought to determine whether chromatin accumulation of the MRN complex in p97-inactivated cells alters 5′-DNA end resection induced by MRE11 nuclease activity. For this, we monitored ssDNA formation by bromodeoxyuridine (BrdU) assay under native conditions. This assay allows visualization of ssDNA by BrdU antibodies in immunofluorescence microscopy (Caron et al., 2019). T24 cells were pretreated with BrdU for 24 h; briefly labeled with 5'-ethynyl-2'-deoxyuridine (EdU), which served as a marker for S-phase cells (EdU-positive cells); and then irradiated or not (Figure 2A). Inactivation of p97 by CB-5083 or siRNA led to increased DNA end resection (ssDNA; BrdU positive) in S-phase (EdU-positive) cells (Figures 2B–2E, S3A, S3B, and S3E), which was reversed upon treatment with the MRE11 inhibitor mirin or by siRNA depletion of MRE11. Cells outside S phase were also investigated, as previous studies have identified that MRE11 also functions in alt-NHEJ in G1 (Biehs et al., 2017). Both treatment with CB-5083 and p97 depletion also caused BrdU foci accumulation outside of S phase (EdU-negative cells; Figures 2F, 2G, and S3C–S3E), which was abrogated in combination with mirin or siRNA-mediated MRE11 depletion. As expected, no BrdU foci formed outside S phase in hydroxyurea (HU)-treated cells, which were used as a positive control of S-phase-dependent DNA end resection. The rescue phenotype due to MRE11 inactivation was also confirmed with two additional MRE11 inhibitors against exonuclease and endonuclease activity, indicating that this is not due to off-target effects of mirin treatment (Figures S4A–S4D). Similar results were obtained when formation of ssDNA (DNA end resection) was analyzed by Replication Protein A (RPA), the major eukaryotic ssDNA-binding protein (Figures 2H and 2I).

A recent report demonstrated that phosphorylated RPA negatively regulates Bloom syndrome helicase and DNA end resection by establishing a negative feedback loop between resection and its termination (Soniat et al., 2019). Therefore, RPA phosphorylation is inhibited in conditions where DNA end resection is hyperactive. To this end, we investigated phosphorylated RPA status in the context of p97 inhibition over 8 h of recovery from IR. We hypothesized that phosphorylated RPA would not accumulate to the same extent as total RPA when DNA end resection is hyperactive. We were able to demonstrate that unlike total RPA (Figures 2H and 2I), fewer serine 4/8 phosphorylated RPA foci were formed in CB-5083-treated conditions after IR than in DMSO-treated cells (Figures 2J and 2K), further supporting our findings that inactivation of p97 causes DNA hyper-resection.

### p97 inhibition delays DSB repair and causes a switch from “error-free” RAD51-mediated repair to mutagenic repair by RAD52

**Figure 3:**
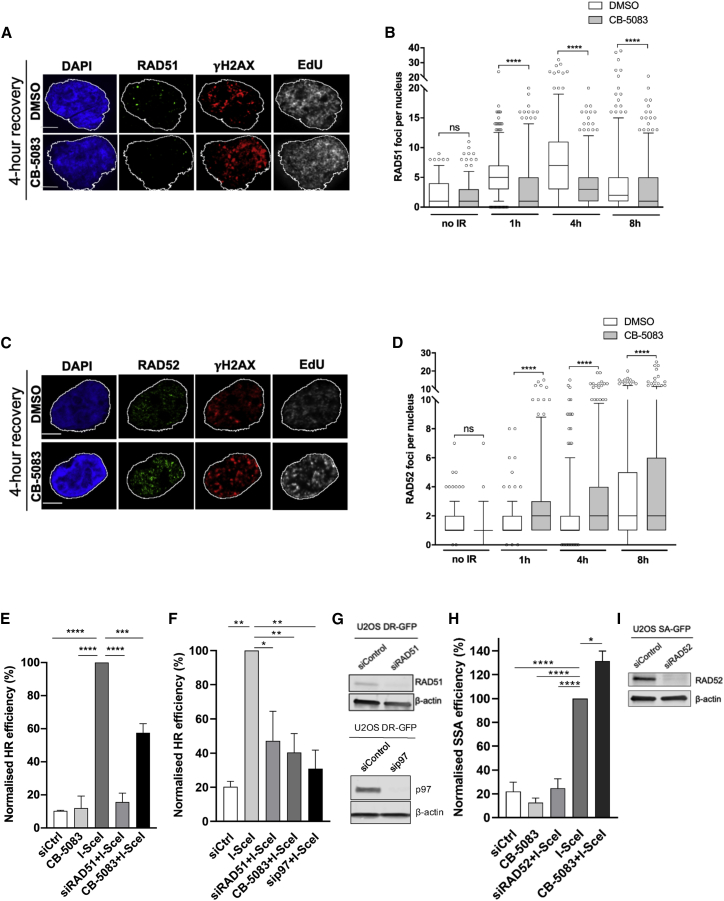
p97 inhibition results in a change from error-free homologous recombination (HR) to mutagenic repair by RAD52 (A and B) Representative images (A) and quantification (B) of the number of RAD51 foci of EdU-positive T24 cells at 4 h after 2 Gy IR and CB-5083 treatment. Scale bars, 5 μm; n = 3. (C and D) Representative images (C) and quantification (D) of the number of RAD52 foci of EdU-positive T24 cells at 4 h after 2 Gy IR and CB-5083 treatment. Scale bars, 5 μm; n = 3. (E) U2OS DR-GFP cells were either transfected with indicated siRNAs for 48 h or treated with 10 μM CB-5083 for 6 h. Cells were transfected with I-SceI plasmid for at least 8 h to induce DNA breaks, and HR repair was monitored by fluorescence-activated cell sorting (FACS). (F) U2OS DR-GFP cells were transfected with I-SceI plasmid as in (E) and treated with p97 siRNA (10 nM over 48 h); n = 3; one-way ANOVA with Dunnett’s multiple comparisons. ^∗^p < 0.05; ^∗∗^p < 0.01; ^∗∗∗^p < 0.001; ^∗∗∗∗^p < 0.0001 (E and F). (G) Western blot showing RAD51 and p97 knockdown efficiencies. (H) U2OS SA-GFP cells were treated as in (E); however, siRAD52 was used instead of siRAD51 as an internal control, and GFP positivity is a readout for SSA repair. The I-SceI group serves as a positive control that is normalized to 100%. One-way ANOVA with Dunnett’s multiple comparisons: ^∗^p < 0.05; ^∗∗∗∗^p < 0.0001; n = 4 replicates. (I) Western blot showing knockdown of RAD52. Data in (B) and (D) are presented as boxplot with interquartile range shown, and values beyond the 5th and 95th percentiles are shown as individual data points. The Mann-Whitney test was used for statistical analysis. NS, not significant; ^∗∗∗∗^p < 0.0001.

When ssDNA levels exceed a certain threshold, HR repair is compromised, and cells are more likely to be repaired by an alternative mutagenic end-joining pathway such as SSA (Duursma et al., 2013). Additionally, compromised 53BP1 recruitment has been shown to cause a switch from error-free gene conversion mediated by RAD51 to RAD52-mediated SSA (Ochs et al., 2016; Jiang et al., 2021). One of the mechanisms by which this occurs is through the release of an inhibitory brake on RAD52 by RAD51 (Ceccaldi et al., 2016). To understand the molecular consequences of increased ssDNA formation after CB-5083 treatment on DNA repair pathway choice, we studied the kinetics of RAD51 (Figures 3A and 3B) and RAD52 (Figures 3C and 3D) foci after 2 Gy IR and observed that p97 inhibition decreases RAD51 foci formation and increases RAD52 foci. To measure DSB repair quantitatively, we used direct-repeat (DR) HR and SSA green fluorescent protein (GFP)-based reporters integrated into U2OS cells, where DSBs are induced by expressing I-SceI nuclease. Inactivation of p97 by either CB-5083 treatment or siRNA depletion caused a reduction in HR-repair efficiency compared to the I-SceI-treated positive control (Figures 3E–3G), indicating that p97 is required for efficient HR repair as previously shown (Meerang et al., 2011; van den Boom et al., 2016). Furthermore, inhibition of p97 increased SSA repair capacity by ∼30% when compared to I-SceI-treated control cells. This indicates that SSA repair is effectively taking place in a background of major HR deficiency (Figures 3H and 3I). Inactivation of RAD51 or RAD52 by siRNA was used as a positive control for defective HR and SSA repair, respectively.

**Figure 4:**
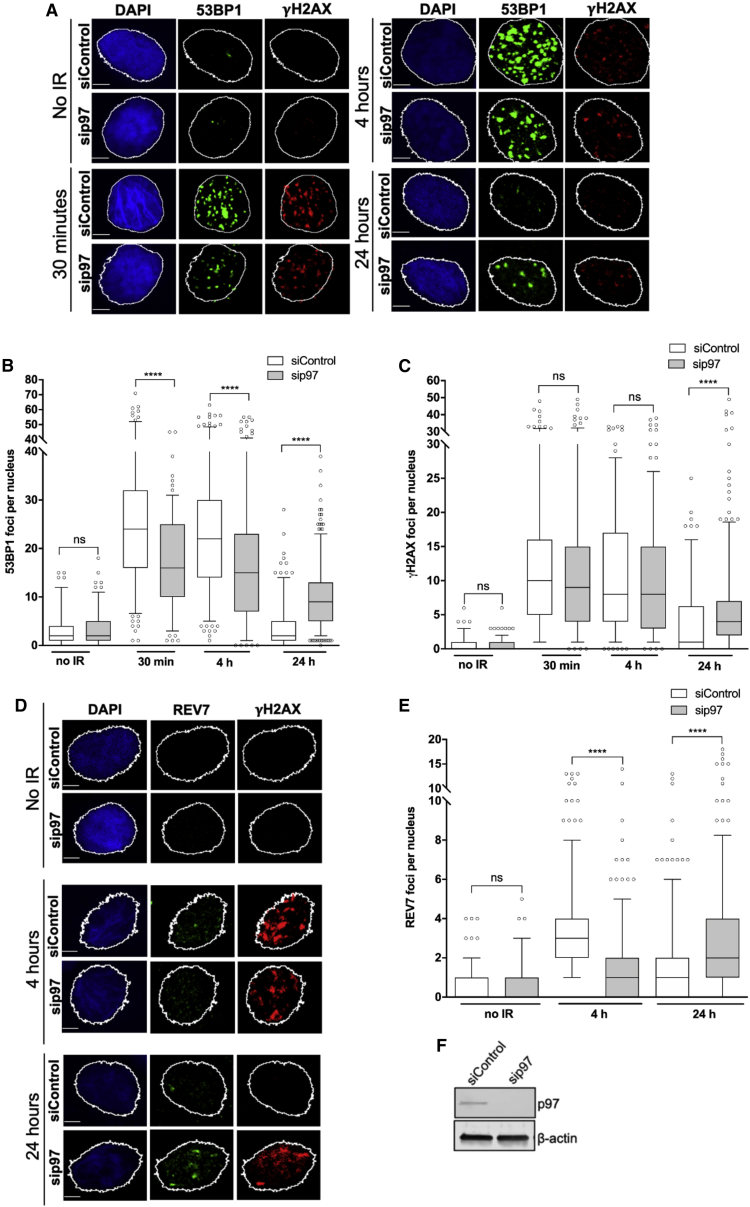
p97 inhibition and depletion negatively regulate the anti-resection factors 53BP1 and REV7, causing a delay in DNA double-strand break (DSB) repair (A–C) Representative images (A) and quantification of 53BP1 (B) and γH2AX (C) foci kinetics over 24 h in p97-depleted T24 cells after 2 Gy IR. Scale bars, 5 μm; n = 3; Mann-Whitney test. NS, not significant; ^∗∗∗∗^p < 0.0001. (D and E) Representative images (D) and quantification of REV7 (E) foci kinetics in p97-depleted T24 cells over 24 h after 2 Gy IR. Scale bars, 5 μm. Data are presented as boxplot with interquartile ranges shown, and values beyond the 5th and 95th percentiles shown as individual points; n = 3; Mann-Whitney test. NS, not significant; ^∗∗∗∗^p < 0.0001. (F) Western blot demonstrating the efficiency of siRNA p97 knockdown. See also Figure S5.

Inactivation of p97 leads to compromised HR activity due to uncontrolled MRE11-dependent DNA end resection. We therefore hypothesized that chemical inhibition of p97 by CB-5083 treatment or siRNA depletion in combination with IR would result in sustained DNA damage and therefore lead to unrepaired DSBs, thus resulting in radiosensitization, which might be exploitable clinically. We quantified 53BP1 and γ-H2AX nuclear foci after 2 Gy IR and monitored repair over 24 h (Figures 4A–4C, 4F, and S5A–S5C). In addition to being a marker of DSB repair efficiency, 53BP1 counteracts DNA end resection by limiting the access of HR factors and nucleases to DNA ends (Ghezraoui et al., 2018). In line with our hypothesis, there was a significant impairment of 53BP1 recruitment in p97-depleted or CB-5083-treated cells until 4 h; however, DNA damage repair was delayed when quantified by the number of γ-H2AX foci over 24 h. Additionally, we questioned whether there is a recruitment defect in other anti-resection factors upon p97 depletion. Specifically, we studied the recruitment of the Shieldin complex component REV7 that is in an effector complex with 53BP1. Depletion of p97 resulted in defective REV7 recruitment to sites of IR-induced damage until 4 h post-IR (Figures 4D–4F), demonstrating similar kinetics to 53BP1. Interestingly, despite defective initial recruitment to the sites of IR-foci in p97-inactivated cells at early time points (over 4 h post-IR), both anti-resection factors (53BP1 and REV7) were more persistent at 24 h post-IR in p97-inactivated cells when compared to control cells. This suggests that DNA damage in p97-inactivated cells persists at 24 h after IR, and some of these damage sites are marked with 53BP1 and REV7. Our conclusion is in accordance with the persistence of γ-H2AX (Figures 4A, 4C, S5A, and S5B) and increased cell sensitivity to IR in p97-inactivated cells (see later in Figure 7).

**Figure 5:**
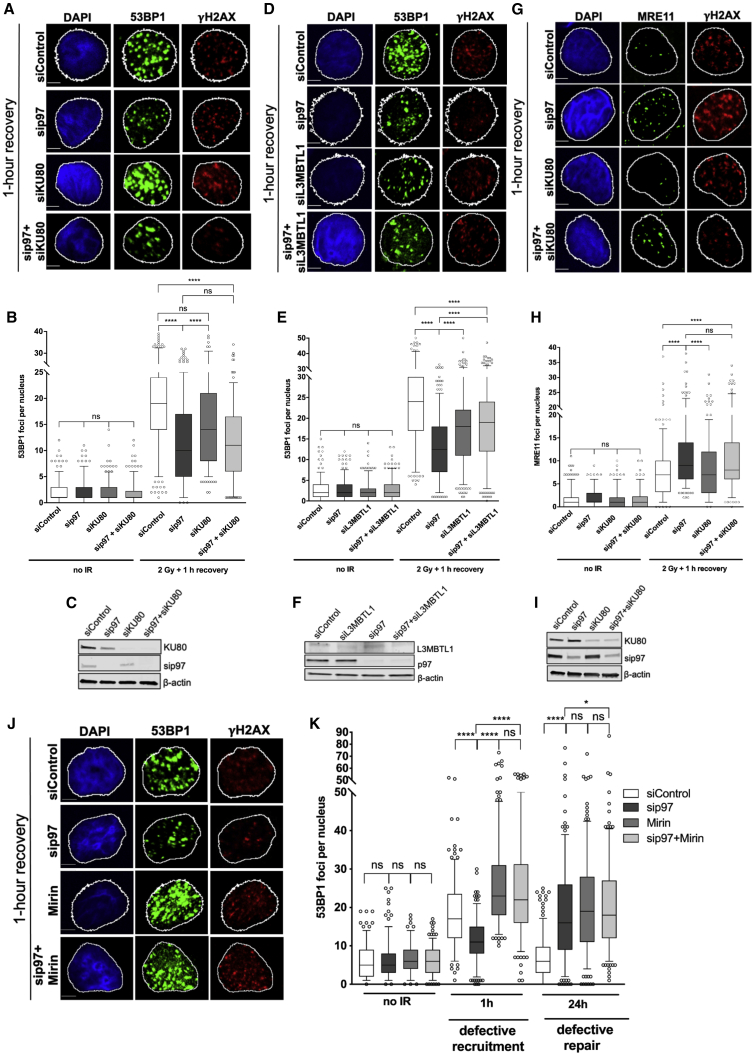
p97 regulates 53BP1 foci formation and accumulation of MRE11 on sites of DNA damage independently of KU70/80 (A and B) Representative images (A) and quantification (B) of the number of 53BP1 foci at 1 h after recovery from 2 Gy IR in T24 cells. (C) Western blot showing knockdown of p97 and KU80. (D and E) Representative images (D) and quantification (E) of the number of 53BP1 foci at 1 h after recovery from 2 Gy IR in T24 cells. (F) Western blot showing knockdown of p97 and L3MBTL1. (G and H) Representative images (G) and quantification (H) of the number of MRE11 foci 1 h after recovery from 2 Gy IR in T24 cells. (I) Western blot showing knockdown of p97 and KU80. (J and K) Representative images (J) and quantification (K) of the number 53BP1 foci at 1 h after recovery from 2 Gy IR in T24 cells. Data in (B), (E), (H), and (K) are presented as boxplot with interquartile ranges shown, and values beyond the 5th and 95th percentiles shown as individual points and analyzed by Mann-Whitney test (B, E, and H) or Kruskal-Wallis test with Dunn’s multiple comparisons (K). NS, not significant; ^∗^p < 0.05; ^∗∗∗∗^p < 0.0001. Scale bars, 5 μm; n = 3. See also Figure S6.

**Figure 6:**
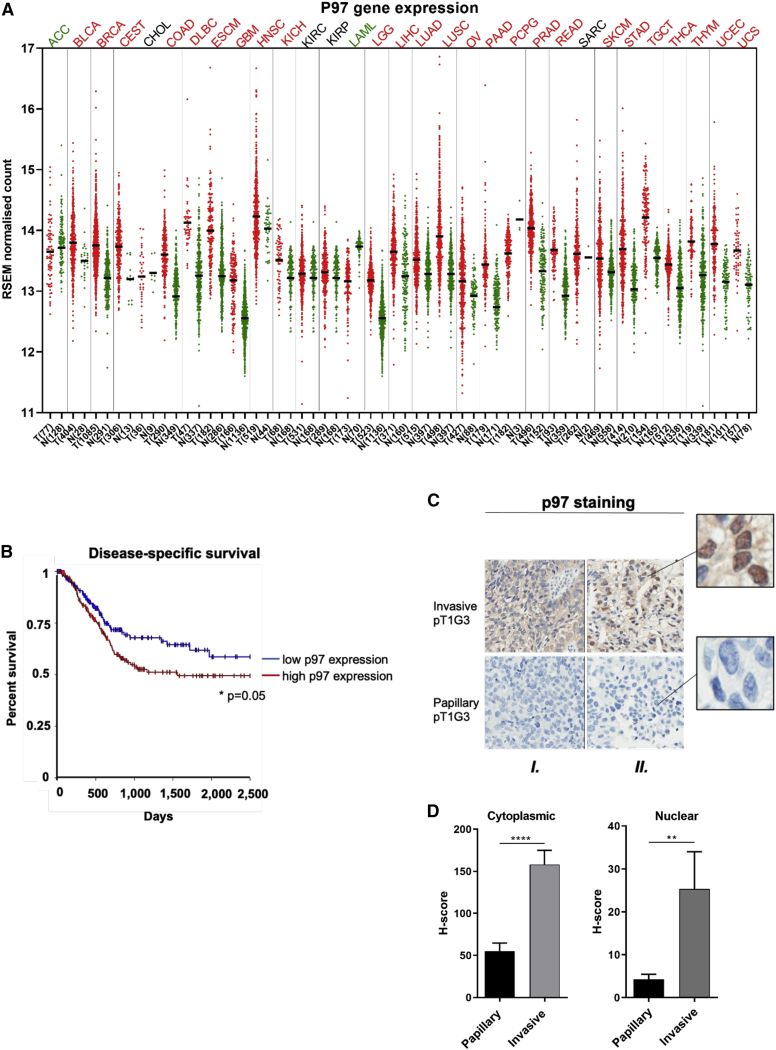
Gene and protein expression levels of p97 in urothelial carcinoma (A) TCGA p97 gene expression levels across different cancer types, comparing tumor expression levels (shown as red dots) to normal tissue (shown as green dots). Bladder cancer is abbreviated “BLCA.” Tumor abbreviations in red have a statistically significant increase in p97 gene expression compared to normal tissues, whereas abbreviations in green have higher levels of p97 gene expression in normal tissues. Abbreviations in black show no statistically significant effect. (B) Disease-specific survival analysis of p97 gene expression; log-rank test, ^∗^p = 0.05. (C) Representative images of two tumor cases from a total of 27 patients with urothelial carcinoma stained with p97 antibody. Each vertical tile represents a separate case (marked as “*I*” and “*II*”). The invasive and papillary areas show the highest and the lowest nuclear staining intensity, respectively. (D) Cytoplasmic and nuclear p97 H-scores in papillary and invasive regions are shown. Data are presented as mean H-score ± SEM. Mann-Whitney *U* test: ^∗∗^p < 0.01 and ^∗∗∗∗^p < 0.0001.

**Figure 7:**
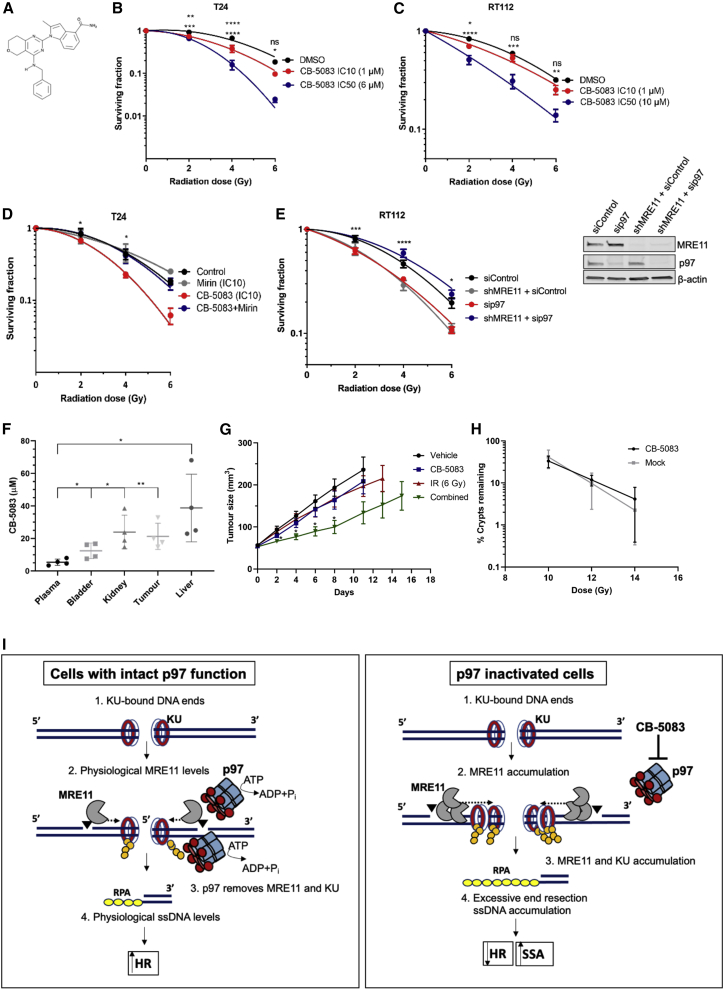
CB-5083 radiosensitizing effects and acute normal tissue toxicity (A) Chemical structure of CB-5083. (B and C) Linear quadratic survival curves of T24 (B) and RT112 (C) cells treated with IR either in combination with CB-5083 IC10 (T24, 1 μM; RT112, 1 μM) and IC50 (T24, 6 μM; RT112, 10 μM), or alone. Statistical significance is indicated on the respective graphs, where the upper row represents IC10 in comparison to the control, and the second row represents IC50 in comparison to the control. NS, not significant; ^∗^p < 0.05; ^∗∗^p < 0.01; ^∗∗∗^p < 0.001; ^∗∗∗∗^p < 0.0001 (two-way ANOVA). See also Figure S2. (D) Survival of T24 cells after IR and treatment with CB-5083, mirin, or both; n = 2 individual biological experiments in triplicate. Error bars represent ± SEM; two-way ANOVA, comparing CB-5083 and CB-5083 + mirin. Statistical significance, ^∗^p < 0.05. (E) Survival of RT112 cells with or without stable small hairpin RNA (shRNA) MRE11 knockdown after treatment with indicated doses of IR. RT112 cells were either treated with siRNA control or siRNA against p97. N = 3 individual biological experiments each over two technical replicates. Error bars represent ± SEM; two-way ANOVA, comparing RT112 + sip97 and RT112 shMRE11 + sip97 conditions. Statistical significance: ^∗^p < 0.05; ^∗∗∗^p < 0.001; ^∗∗∗∗^p < 0.0001. Right: western blot of the experiment in (E). (F) Plasma, bladder, kidney, liver, and tumor concentrations (in μM) of CB-5083 in tissue extracts from CD-1 nude mice bearing subcutaneous RT112 tumors after treatment at 25 mg/kg (N = 5). Statistical analysis: ^∗^p < 0.05; ^∗∗^p < 0.01 (unpaired t test). (G) RT112 cells were subcutaneously injected into CD-1 nude mice to establish tumors. Tumor growth was measured after a single intraperitoneal injection of 25 mg/kg CB-5083 and radiotherapy (6 Gy, single fraction) in RT112 xenografts (N = 8 per group). ^∗^p < 0.05 (one-way ANOVA with Dunnett’s multiple comparisons). (H) Small crypt assay survival for CB-5083 and mock-treated C-1 nude mice over 10 Gy (N = 3 per group), 12 Gy (N = 6 per group), and 14 Gy (N = 3 per group) of IR. Data were normalized to mean crypts per millimeter in mock-treated and CB-5083-only-treated mice. (I) Model of the role of p97 in DSB repair pathway choice. Left: under physiological conditions, both MRE11 and the KU complex are removed from the sits of DSBs by the ATPase activity of p97 to promote HR-mediated repair. Right: inactivation of p97 causes accumulation of MRE11 and the KU complex on the sites of DSBs, leading to excessive 5′-DNA end resection, subsequent HR defects, and an increase in mutagenic SSA-mediated repair. See also Figure S7.

To understand whether the 53BP1 recruitment defect observed in p97 inactivated cells is a direct result of MRE11 regulation or an indirect effect, we studied 53BP1 recruitment to sites of IR-induced DNA damage in both KU80- and Lethal(3)malignant brain tumor-like protein 1 (L3MBTL1)-depleted conditions. KU80 and L3MBTL1 are known p97 substrates upstream of 53BP1 and REV7, affecting DSB repair pathway choice (van den Boom et al., 2016; Acs et al., 2011). p97-dependent removal of KU80 directs the DSB repair pathway from NHEJ to HR by facilitating long-range DNA end resection, and p97-dependent removal of L3MBTL1 facilitates the recruitment of 53BP1 to methylated histone H4 in the vicinity of DNA damage. Depletion of KU80 did not rescue the 53BP1 recruitment defect in p97-depeleted cells (Figures 5A–5C), indicating that p97 regulates 53BP1 independently of the KU70/80 heterodimer. Additionally, we observed only a partial rescue of 53BP1 foci recruitment in p97-depleted cells when L3MBTL1 was co-depleted (Figures 5D–5F, S6A, and S6B), indicating that L3MBTL1 is not the sole p97 substrate involved in controlling 53BP1 recruitment to sites of DSBs. This suggests that other p97 substrates upstream of 53BP1 most likely influence 53BP1 recruitment to IR-induced foci. We also confirmed that p97 controls MRE11 chromatin accumulation independently of KU70/80, as increased accumulation of MRE11 at sites of DNA damage/IR-induced foci in p97-depleted cells was not abolished by KU80 co-depletion (Figures 5G–5I).

We next sought to understand whether a defect in 53BP1 recruitment to sites of DNA damage in p97-inactivated cells could be rescued by inactivation of the MRE11 nuclease activity. To do so, T24 cells were treated with mirin, and the recruitment of 53BP1 to the sites of IR-induced foci/DNA damage was monitored. Interestingly, mirin-treated cells were able to fully rescue defective 53BP1 recruitment in p97-depleted cells (Figures 5J and 5K), indicating that the MRE11 nuclease activity poses a barrier to 53BP1 recruitment when p97 is inactivated. Additionally, there was no additive effect on 53BP1 recruitment in p97-depleted cells when L3MBTL1 was co-depleted in combination with mirin (Figures S6C and S6D). Overall, these results demonstrate that p97 regulates MRE11 independently of the KU70/80 complex but also 53BP1 recuitment by preventing excessive MRE11-dependent end resection.

### p97 is more highly expressed in invasive than papillary bladder cancer

Previous studies have shown p97 overexpression in several tumors (Tsujimoto et al., 2004; Yamamoto et al., 2004a, 2004b, 2004c) and its association with metastasis and poor prognosis in cancer patients (Tsujimoto et al., 2004). Using The Cancer Genome Atlas (TCGA) data (Tang et al., 2019), we found statistically significantly higher p97 gene expression in breast, colon, and prostate cancer compared to equivalent normal tissues (Figure 6A). p97 gene expression levels were also significantly higher in muscle invasive bladder cancer (tumors N = 404) compared to only 28 normal tissue samples. In the bladder cancer cases, high tumor p97 expression levels were significantly correlated with poorer disease-specific survival following bladder removal by cystectomy (Figure 6B).

Using immunohistochemistry, we compared cytoplasmic and nuclear protein expression of p97 in papillary and invasive tumor areas by H-score in 27 patient samples with high-grade non-muscle invasive bladder cancer (NMIBC) with invasion to the lamina propria (HGT1 stage). Strong cytoplasmic and nuclear p97 staining was observed in invasive compared to papillary areas (Figures 6C and 6D), suggesting that pathological progression of bladder cancer results in cells becoming more dependent on p97 function for survival.

### CB-5083 acts as a radiosensitizer in bladder cancer

Inhibition of p97 has been found to induce apoptosis and reduce overall cell survival in several cancer cell lines and mouse solid tumor models (Anderson et al., 2015; Zhou et al., 2015; Le Moigne et al., 2017; Auner et al., 2013). Furthermore, p97 depletion has been found to sensitize U2OS cells to IR (Meerang et al., 2011). We investigated p97 as a target for radiosensitization using T24 and RT112 bladder cancer cell lines in a clonogenic assay, in the presence or absence of CB-5083, at inhibitory concentration (IC)10 and IC50 doses delivered 6 h prior to IR (Figures 7A–7C, S7A, and S7B). CB-5083-treated cells achieved significant radiosensitization compared to control cells, as shown by reduced clonogenic survival (Figures 7B and 7C). Supportive of our molecular findings *in vitro*, where MRE11 nuclease inhibition by mirin completely rescued the p97 inhibition phenotype, we found in clonogenic assays that mirin treatment rescued radiosensitization caused by CB-5083 (Figures 7D and S7C). To further support this finding, we confirmed the aforementioned rescue phenotype by depleting p97 in RT112 cells with a stable small hairpin RNA (shRNA) knockdown of MRE11 (shMRE11; Figure 7E). As predicted, either siRNA-mediated p97 depletion or shRNA MRE11 knockdown resulted in IR hypersensitivity of RT112 cells (Singh et al., 2019; Meerang et al., 2011; Xu et al., 2004; Yamaguchi-Iwai et al., 1999; Na et al., 2021; Stracker and Petrini, 2011). Importantly, co-depletion of p97 and MRE11 by siRNA and shRNA, respectively, completely rescued IR sensitivity of RT112 cells. We conclude this rescue effect is due to suppression of excessive MRE11-dependent DNA end resection in p97-inactivated cells (Figures 2A–2G, S3, and S4) and consequent restoration of 53BP1 foci (Figures 5J–5K) that promote efficient NHEJ repair, the main pathway for cell survival upon IR-induced DNA damage. Altogether, our results suggest that one of the main roles of p97 in DDR and cell survival upon IR is the regulation of MRE11 protein level on sites of DNA damage and consequently its nuclease activity.

An alternative treatment to bladder removal by cystectomy in muscle-invasive bladder cancer (MIBC) is bladder preservation, based on treatment with radiotherapy (Kiltie, 2014; Alfred Witjes et al., 2017). As many currently used radiosensitizing agents add to the toxicity of radiotherapy, new approaches are required. Proteotoxic stress is a secondary hallmark of cancer (Guang et al., 2019), because cancer cells accumulate defective proteins at a significantly higher rate compared to normal cells, which makes cancer cells more dependent on the proteasome for their removal (Adams, 2004). Therefore, the use of CB-5083 may spare normal tissues.

CB-5083 has been studied as a single agent *in vivo*, but not in combination with IR. Having established CB-5083 as a radiosensitizer in bladder cancer cell lines, we used a bladder cancer superficial flank xenograft model to determine whether inhibiting p97 would sensitize tumors to IR. CD-1 nude mice bearing RT112 xenografts were treated with a single dose of CB-5083 at 25 mg/kg via intraperitoneal injection, and plasma, liver, kidney, bladder, and tumor concentrations of CB-5083 were analyzed 6 h after treatment to determine CB-5083 uptake in various organs and in tumors (Figure 7F). Uptake was significantly higher in tumors (mean, 25 μM) than the CB-5083 plasma concentration (mean, 5 μM). Additionally, CB-5083 uptake was significantly higher in the organs analyzed compared to plasma. The drug was well tolerated at these doses, with no significant body weight loss over the course of a week in CD-1 nude mice given a single intraperitoneal dose of CB-5083 at 25 mg/kg (n = 2) or 50 mg/kg (Figure S7D).

Mice with RT112 xenograft tumors treated with a single 25-mg/kg intraperitoneal injection of CB-5083 6 h prior to radiotherapy (6 Gy, single fraction) showed significant tumor growth inhibition at day 8 compared to control, IR-only, or drug-only groups (Figure 7G). Although the time to reach 50 mm^3^ in size for the start of the treatment was not significantly different between groups (Figure S7E), the time to treble tumor volume was significantly longer in the combined group than in the control group, but not in the IR-only and CB-5083-only groups (Figure S7F).

We then studied the effects of an intraperitoneal injection of 25 mg/kg CB-5083 on acute intestinal toxicity from IR in CD-1 nude mice using an intestinal crypt assay as previously described (Groselj et al., 2018). The vehicle- and CB-5083-alone groups demonstrated no overall crypt loss, and CB-5083 in combination with 10, 12, or 14 Gy radiation demonstrated no further loss of crypts compared to radiation alone (Figures 7H and S7G). Altogether, these *in vivo* results suggest that a single dose of the p97 inhibitor CB-5083 at 25 mg/kg reduces the growth of bladder cancer xenografts treated with IR, with no exacerbation of acute normal tissue toxicity to the surrounding small intestine of CD-1 nude mice under the observed conditions. These *in vivo* data support our biochemical and cell biological data obtained *in vitro*.

## Discussion

We demonstrate that MRE11 accumulation on DNA damage and chromatin is regulated by the ubiquitin-dependent ATPase p97 and show that inhibiting p97 activity leads to uncontrolled MRE11 nuclease activity on chromatin. However, maintaining balanced levels of MRE11 on chromatin is necessary for genomic stability. We postulate that disrupting this balance by inactivating p97 could be one of the mechanisms by which cancer cells are radiosensitized and are therefore more likely to undergo cell death. We propose this occurs because excessive accumulation of MRE11 on chromatin leads to pathological 5′-DNA end resection and inhibition of the error-free HR pathway. Our model (Figure 7I) has potential implications for cancer therapy.

p97 is a molecular segregase involved in the timely removal of DDR factors to maintain genomic stability. Among its known substrates at sites of DNA damage are key DNA repair factors such as the KU complex, RNF8, and RAD51 (Singh et al., 2019; Torrecilla et al., 2017). Our mass spectrometry data, confirmed by chromatin immunoprecipitation, identified that the MRN complex is one of the most prominent complexes associated with p97, where the number of p97 peptides in complex with MRE11 was similar to the number of the two MRE11 constitutive binding partners RAD50 and NBS1. This indicates that p97 might be one of the essential factors for the function of the MRN complex and thus important for controlling MRE11 nuclease activity. To address the functional relevance of the interaction between p97 and the MRN complex, we demonstrate that p97 inactivation blocks the disassembly of the MRN complex from chromatin, especially its catalytic subunit, MRE11, but does not affect the overall turnover of the MRN complex. This has important implications for DNA repair and cell survival.

Homeostasis of the nuclease MRE11 is necessary in the maintenance of genomic stability, as insufficient MRE11 nuclease activity compromises error-free HR repair (Jachimowicz et al., 2019; Buis et al., 2008). Furthermore, its over-accumulation leads to excessive DNA end resection that results in pathological DNA repair, degradation of nascent DNA strands, and genome instability syndromes (Hashimoto et al., 2010; Ray Chaudhuri et al., 2016; Schlacher et al., 2011). Previously, it has been shown that UBQLN4 removes ubiquitinated MRE11 from chromatin to favor NHEJ (Jachimowicz et al., 2019), DYNLL1 interacts with MRE11 directly to limit its nuclease activity in BRCA1-deficient cells (He et al., 2018), and C1QBP binds directly to MRE11 to maintain the MRE11/RAD50 protein pool while limiting MRE11 exonuclease activity (Bai et al., 2019). Furthermore, ataxia telangiectasia mutated (ATM)-dependent phosphorylation of MRE11 at S676/678 leads to its removal from chromatin, and phospho-defective mutations of these two serines cause MRE11 retention on chromatin, excessive DNA end resection, and decreased HR but a functional SSA repair pathway (Kijas et al., 2015). However, how exactly MRE11 is disassembled from the sites of DNA damage is poorly understood.

Here, we demonstrate that the ATPase activity of p97 physically disassembles MRE11 from the sites of DNA damage. Thus, p97 inactivation has functional relevance for MRE11 nuclease activity by causing excessive ssDNA formation. It has been previously shown that when DSBs accumulate ssDNA above physiological levels, HR is compromised and cells are more likely to be repaired by an alternative and mutagenic SSA pathway (Duursma et al., 2013). For example, it is known that upon compromising 53BP1 foci formation, DNA repair template switch occurs from RAD51-mediated HR to RAD52-mediated SSA (Ochs et al., 2016). This has pathological consequences for the cell, as it leads to a change from error-free repair to mutagenic repair, causing deletion rearrangements within the genome (Bhargava et al., 2016). In line with previous literature, we also demonstrate that inactivating p97 leads to reduced Rad51 foci formation after IR and a template switch from RAD51-mediated HR to SSA mediated by RAD52. The reason for the switch from RAD51 to RAD52 occurs because of their antagonistic relationship, and upon disruption of RAD51 function, an inhibitory brake on RAD52 is released (Ceccaldi et al., 2016). Furthermore, decreased recruitment of the anti-resection factors 53BP1 and REV7 to the sites of IR-induced foci in p97-inactivated cells forms a permissive environment for DNA end resection, which is in agreement with our observations.

It has been shown that p97-dependent disassembly of the KU70/80 heterodimer from DSB ends is essential for DSB repair and regulation of HR-repair pathway (van den Boom et al., 2016). Specifically, inactivation of the p97-Ufd1-Npl4 complex causes retention of the KU70/80 heterodimer, which stimulates NHEJ repair but blocks the HR-repair pathway. This might suggest that the here-discovered regulation of MRE11 by p97 is an indirect process over KU70/80. However, we demonstrated that regulation of MRE11 by p97 is a direct process, as p97 physically forms a complex with the MRN complex, and MRE11 retention on sites of DNA damage in p97-inactivated cells is KU70/80 independent. Also, the 53BP1 recruitment defect seen in p97-inactivated cells could not be restored by KU80 depletion, which causes degradation of the entire KU70/80 complex (van den Boom et al., 2016; Nussenzweig et al., 1996; Fink et al., 2010). Conversely, the 53BP1 foci recruitment defect in p97-inactivated cells could be completely or partially restored by inactivation of MRE11 by mirin or depletion of L3MBTL1, respectively. The full restoration of 53BP1 IR foci in p97-inactivated cells by MRE11 inactivation, either by chemical inhibition (mirin) or by siRNA-mediated MRE11 depletion, in two different cell lines indicates that MRE11 accumulation acts as a barrier to 53BP1 recruitment in p97-inactivated conditions. Altogether, our findings demonstrate that the physical and functional relationship between p97 and MRE11 is a separate process from the previously described p97-KU70/80 axis in the regulation of DSB pathway choice.

After understanding the molecular aspects of p97 inactivation, we demonstrated the potential clinical benefits of p97 inhibition in combination with radiotherapy. When cancer cells become dependent on the UPS for survival, it is expected that targeting specific agents within the UPS will lead to cancer cell death while having minimal effects on normal cells, as there is no proteotoxic stress under normal conditions (Adams, 2004). By combined targeting of proteostasis and the DDR, we validated the usefulness of combined p97 inhibitor/radiotherapy using clonogenic assays and a mouse xenograft model. With no increase in acute radiation toxicity following addition of CB-5083 at a dose that achieved tumor radiosensitization, targeting proteotoxic stress may be a promising clinical target. Furthermore, by assessing p97 expression in NMIBC (HGT1) patient samples, we demonstrate that both cytoplasmic and nuclear p97 expression levels are significantly higher in more invasive areas compared to papillary areas. This is in agreement with previous literature showing that overexpression of p97 occurs in a variety of tumor types (Tsujimoto et al., 2004; Yamamoto et al., 2004a, 2004b, 2004c), and this overexpression exemplifies the need for the UPS in cancer cells as a survival mechanism. Additionally, we validated these results using the TCGA database on p97 gene expression levels in various tumors compared to normal tissues. All of the above observations support the notion that cancer cells rely on the function of p97 for survival.

In summary, we have shown that by controlling the timely removal of MRE11 from chromatin, p97 regulates the extent and balance of DNA end resection after DNA damage. By inactivating p97, we show that MRE11 accumulates on chromatin, which leads to ssDNA buildup in cells. The MRE11-mediated excessive end resection decreases HR efficiency and increases RAD52-dependent SSA. Consequently, this leads to increased tumor cell killing following IR both *in vitro* and *in vivo*. We propose this could be therapeutically exploited in further studies.

## STAR★Methods

### Key resources table

**Table:** 

REAGENT or RESOURCE	SOURCE	IDENTIFIER
**Antibodies**		

BrdU Mouse monoclonal	BD Biosciences	Cat# 347580; RRID:AB_10015219
Phospho-Histone H2A.X (Ser139) Mouse monoclonal	Millipore	Cat#05-636; RRID:AB_309864
Phospho-Histone H2A.X (Ser139) Rabbit polyclonal	Cell Signaling	Cat# 2577; RRID:AB_2118010
RPA70/RPA1 Rabbit polyclonal	Cell Signaling	Cat# 2267; RRID:AB_2180506
P97/VCP Rabbit polyclonal	Proteintech	Cat# 10736-1-AP; RRID:AB_2214635
53BP1 Rabbit polyclonal	Cell Signaling	Cat# 4937; RRID:AB_10694558
RAD51 Mouse monoclonal	Santa Cruz	Cat# 398587; RRID:AB_2756353
RAD52 Mouse monoclonal	Santa Cruz	Cat# 365341; RRID:AB_10851346
RAD50 Mouse monoclonal	Abcam	Cat# 89; RRID:AB_2176935
MRE11 Rabbit polyclonal	Abcam	Cat# 33125; RRID:AB_776530
MRE11 Mouse monoclonal	Abcam	Cat# ab214; RRID:AB_302859
L3MBTL1 Rabbit polyclonal	Abcam	Cat# ab51880; RRID:AB_873913
MAD2B Mouse monoclonal	BD Biosciences	Cat# 612266; RRID:AB_399583
NBS1 Mouse monoclonal	BD Biosciences	Cat# 611870; RRID:AB_399350
Vinculin Mouse monoclonal	Abcam	Cat# ab18058; RRID:AB_444215
Lamin A/C Mouse monoclonal	Cell Signaling	Cat# 4777; RRID:AB_10545756
Histone H2B Rabbit monoclonal	Cell Signaling	Cat# 12364; RRID:AB_2714167
Alexa Fluor 488 Rabbit polyclonal	Thermo Fisher Scientific	Cat# A-11034; RRID:AB_2576217
Alexa Fluor 488 Mouse polyclonal	Thermo Fisher Scientific	Cat# A-21202; RRID:AB_141607
Alexa Fluor 568 Rabbit polyclonal	Thermo Fisher Scientific	Cat# A-11011; RRID:AB_143157
Alexa Fluor 568 Mouse polyclonal	Thermo Fisher Scientific	Cat# A-10037; RRID:AB_2534013
RPA32/RPA2 (phospho S4+S8) rabbit polyclonal	Abcam	Cat# ab87277; RRID:AB_1952482
KU80 Mouse monoclonal	Abcam	Cat# 119935; RRID:AB_10899161
Lamin B1 Rabbit polyclonal	Thermo Fisher Scientific	Cat# PA5-19468; RRID:AB_10985414
Mouse 800 secondary	LI-COR	Cat# 925-32210; RRID:AB_2687825
Rabbit 680 secondary	LI-COR	Cat# 925-68021; RRID:AB_2713919
β-actin Mouse monoclonal	Thermo Fisher Scientific	Cat# AM4302; RRID:AB_437394
β-actin Rabbit (13E5) monoclonal	Cell Signaling	Cat# 4970; RRID:AB_2223172
Control Rabbit IgG	Non-commercial	Kristijan Ramadan Lab
Rabbit IgG (HRP conjugated) Goat polyclonal	Sigma-Aldrich	Cat# A9169; RRID:AB_258434
Mouse IgG (HRP conjugated) Rabbit polyclonal	Sigma-Aldrich	Cat# A9044; RRID:AB_258431

**Biological samples**		

Bladder cancer human tumor samples (HGT1)	Oxford Radcliffe Biobank	http://orb.ndcls.ox.ac.uk

**Chemicals, peptides, and recombinant proteins**		

BrdU	Sigma-Aldrich	Cat# B5002
EdU	Thermo Fisher Scientific	Cat# A10044
Cycloheximide	Sigma-Aldrich	Cat# C1988
Benzonase	Millipore	Cat# 71205
Doxycycline	PanReac AppliChem	Cat# A2951,0025
Hoechst 3325	Sigma-Aldrich	Cat# 23491-45-4
RNase A (10 mg/mL)	Thermo Fisher Scientific	Cat# EN0531
ProLong Diamond antifade with DAPI	Thermo Fisher Scientific	Cat# P36962
cOmplete, Mini, EDTA-free Protease Inhibitor Cocktail	Roche	Cat# 11836170001
Intercept® (PBS) Blocking Buffer	LI-COR	Cat# 927-70001
Sequencing Grade Modified Trypsin	Promega	Cat# V5111
BSA	Sigma-Aldrich	Cat# 9048-46-8
Fisher Bioreagents Phosphatase Inhibitor Cocktail I	Thermo Fisher Scientific	Cat# 12821650
Pierce Protease Inhibitor Mini Tablets, EDTA-free	Thermo Fisher Scientific	Cat# A32955
Hoechst 33342	Thermo Fisher Scientific	Cat# H3570
Lipofectamine RNAiMAX	Thermo Fisher Scientific	Cat# 13778-150
QIAGEN maxiprep kit	QIAGEN	Cat# 12362
FuGENE Transfection Reagent	Promega	Cat# E2311
jetPRIME Transfection Reagent	Polyplus-transfection	Cat# 114-01
Opti-MEM reduced serum medium	Thermo Fisher Scientific	Cat# 11058021
CB-5083	MedChem Express	Cat# HY-12861
Mirin	Sigma-Aldrich	Cat# M9948
PFM01	Tocris	Cat# 6222
PFM39	Sigma-Aldrich	Cat# SML1839
DMSO	Sigma-Aldrich	Cat# D2650
Methyl cellulose	Thermo Fisher Scientific	Cat# 155496
NEM	Sigma-Aldrich	Cat# EE3876-25G
Tween-20	Sigma-Aldrich	Cat# P7949
Tween-80	Sigma-Aldrich	Cat# 9005-65-6
Puromycin	GIBCO	Cat# A11138-03
G-418	Sigma-Aldrich	Cat# 108321-42-2
Gefitinib	Sigma-Aldrich	Cat# SML1657
Matrigel	BD Biosciences	Cat# 356237
PBS	GIBCO	Cat# 10010023
20X Transfer buffer	Bio-Rad	Cat# 10026938
10X Running buffer	Bio-Rad	Cat# 1610772
Laemmli 2X concentrate	Sigma-Aldrich	Cat# S3401
Antibody diluent	LI-COR	Cat# 927-75001
Hydroxyurea (HU)	Sigma	Cat# H8627
Triton X-100	Sigma-Aldrich	Cat# T8787
HEPES	Sigma-Aldrich	Cat# H4034
Sucrose	Sigma-Aldrich	Cat# S0389
MgCl_2_	Thermo Fisher Scientific	Cat# AM9530G
NaCl	Sigma-Aldrich	Cat# S5150
EDTA	Thermo Fisher Scientific	Cat# 15575020
Tris hydrochloride	Sigma-Aldrich	Cat# 1185-53-1
Formaldehyde 36% (39% w/v)	VWR	Cat# 50-00-0
Click-IT EdU Alexa Fluor 647 imaging kit	Thermo Fisher Scientific	Cat# C10340
Pierce Anti-HA Magnetic Beads	Thermo Fisher Scientific	Cat# 88836
Dynabeads Protein A	Thermo Fisher Scientific	Cat# 10002D
Portein A magnetic beads	NEB	Cat# S1425S
Dynabeads Protein G	Thermo Fisher Scientific	Cat# 10003D
Strep-Tactin® Sepharose® 50% suspension beads	IBA Life Sciences	Cat# 2-1201-010
MagStrep “type3” XT beads	IBA Life Sciences	Cat# 2-4090-002

**Deposited data**		

ProteomeXchange Consortium	PRIDE	PXD016279

**Experimental models: cell lines**		

Human: T-24	German Collection of Microorganisms and Cell Cultures (DSMZ)	DSMZ No 376
Human: RT112	German Collection of Microorganisms and Cell Cultures (DSMZ)	DSMZ No 418
Human: HEK293	ATCC	Cat# 300192/p777_HEK293
Human: HeLa	ATCC	Cat# CCL-2
Human: DR-GFP U2OS	Laboratory of Dr Timothy Humphrey (Ahrabi et al., 2016)	N/A
Human: SA-GFP U2OS	Laboratory of Dr. Pavel Janscak (Paliwal et al., 2014)	N/A
HEK293 Flp-In T-REx (p97-E578Q-cSSH)	Laboratory of Prof Kristijan Ramadan (Meerang et al., 2011)	N/A
RT112 (shMRE11)	Laboratory of Prof Anne E Kiltie (Na et al., 2021)	N/A

**Experimental models: organisms/strains**		

CD-1 nude mice (Female)	Charles River Laboratories	Strain Code: 086 (Homozygous)

**Oligonucleotides**		

siRNA_Control (proprietary sequence)	QIAGEN	Cat# 1027280
siRNA_RAD51 (5′- AAGGGAATTAGTGAAGCCAAA-3′)	QIAGEN	Cat# SI02663682
siRNA_RAD52 (5′-AAGGATGGTTCATATCATGAA-3′)	QIAGEN	Cat# SI03035123
siRNA_MRE11 (5′GAGCAUAACUCCAUAAGUA(dT)(dT)-3′)	Thermo Fisher Scientific	Cat# 10620310
siRNA_XRCC5/siKU80 (5′-GCAUGGAUGUGAUUCAACA-3′)	Dharmacon	Cat# 7520
siRNA_VCP_7 (5′-UUCUGUUUGAGAAUGGCUGUU-3′)	Thermo Fisher Scientific	Cat# 10620310
siRNA_VCP_7 (5′-AACAGCCAUUCUCAAACAGAA-3′)	QIAGEN	N/A
siRNA_L3MBT1_5 (5′-AAGCGAGUAUAAGCC-3′)	Microsynth	N/A
siRNA_L3MBT1_7 (5′-CCAGGCAGUCACUCA-3′)	Micorynth	N/A

**Recombinant DNA**		

pcDNA3.3 N-term HA-MRE11	Laboratory of Anne E Kiltie (Nicholson et al., 2017)	N/A
pcDNA5-p97-EQ-Myc-Strep	Laboratory of Hemmo Meyer or Kristijan Ramadan (Meerang et al., 2011)	N/A

**Software and algorithms**		

Prism 8	GraphPad Software	https://www.graphpad.com
Fiji	NIH	https://imagej.net/Fiji/Downloads
FlowJo v10	FlowJo	https://www.flowjo.com/solutions/flowjo/downloads
Image Studio Lite 5.2	LI-COR	https://www.licor.com/bio/image-studio-lite/download
UCSC Xena	University of California, Santa Cruz	https://www.biorxiv.org/content/10.1101/326470v6
Proteome Discoverer Software	Thermo Fisher Scientific	https://thermo.flexnetoperations.com/control/thmo/login
MASCOT 2.3	Matrix Science Inc	http://www.matrixscience.com/
PRIDE	Wellcome Genome Campus, Hinxton, Cambridgeshire	https://www.ebi.ac.uk/pride/archive/

### Resource availibility

#### Lead contact

Kristijan Ramadan (kristijan.ramadan@oncology.ox.ac.uk) should be contacted for requests regarding resources, data and reagents used in this study.

#### Materials availability

All reagents generated in this study are available from the lead contact or Anne E Kiltie, except human HGT1 tissue sections. The Oxford Radcliffe Biobank (http://orb.ndcls.ox.ac.uk) should be contacted for HGT1 tissue sections.

#### Data and code availability

MRE1 -mass spectrometry proteomics data have been deposited to the ProteomeXchange Consortium via the PRIDE partner repository with the dataset identifier PXD016279**.**

### Experimental model and subject details

#### Mice

All animal experiments were conducted in accordance with Home Office regulations for animal testing and research and approved by the University of Oxford Animal Welfare and Ethical Review Body (AWERB) under University of Oxford project licenses PPL P4B738A3B and P8484EDAE. CD-1 nude mice were obtained from Charles River Laboratories at the age of 7 weeks. All mice used were female and the experiment started when the mice were at the age of 8 weeks. Mice were weighed before the start of experiment and from there onward three times a week. Mice were kept up to 6 mice per cage and maintained in individually ventilated cages (IVCs).

#### Human HGT1 tissue sections

High-grade non-muscle invasive bladder cancer human tumor samples (HGT1) were identified in the Oxford Radcliffe Biobank. Twenty-seven cases obtained between the years 2009 to 2015 were used under the appropriate ethical approval (South Central Oxford REC C 09/H0606/5+5). A total of eight patients underwent radical cystectomy. Muscle invasive disease was identified in the resection specimen of one patient post-cystectomy (pT2). Two patients demonstrated disease progression following biopsy for disease recurrence in 2013 and 2015. Both patients included in Figures 6C and 6D (I and II) were males, and 59 and 69 years old, respectively.

#### Cell lines

T24 and RT112 cell lines were obtained from German Collection of Microorganisms and Cell Cultures (DSMZ). HeLa and HEK293 cell lines were obtained from the American Type Culture Collection (ATCC). All cell lines were grown in complete medium supplemented with 10% FBS.

### Method details

#### Cell culture

T24 and HEK293 cells were grown in DMEM medium, and RT112 cells were grown in RPMI-1640 medium. DR-GFP U2OS cells were provided by Dr. Sovan Sarkar, University of Oxford, and grown in DMEM medium supplemented with 3 μg/mL puromycin. SA-GFP U2OS cells were provided by Dr. Pavel Janscak, University of Zurich, and grown in DMEM supplemented with 3 μg/mL puromycin. Stable HEK293 Flp-In T-REx (p97-E578Q-cSSH) cells for p97 SILAC-MS were used as previously described (Meerang et al., 2011). RT112 cells with stable shRNA knockdown of MRE11 were kept in RPMI-1640 medium supplemented with G418 (500 μg/mL), and provided by Dr. Juri Na, University of Oxford. Cells were treated as follows: 10 μM BrdU (#B5002; Sigma) and 10 μM Edu (#C1042; Sigma) for 24 hours and in the last 20 minutes of the 24 hours, respectively; 10 μM CB-5083 (#HY-12861; MCE) for 2 hours; 500 μM hydroxyurea (#H8627; Sigma) for 1 hour; MRE11 inhibitors used were 100 μM Mirin (#M9948; Sigma), 100 μM PFM01 (#6222; Tocris), 100 μM PFM39 (#SML1839; Sigma) for 3 hours. Concentrations of PFM01 and PFM39 were chosen according to a previous publication (Shibata et al., 2014). For clonogenic survival assays, appropriate concentrations of CB-5083 and Mirin were applied for 6 hours and 24 hours, respectively.

#### RNA interference

The siRNA sequences for control and indicated genes were transfected in cells using Lipofectamine RNAiMAX (Thermo Fisher Scientific) according to the manufacturer’s protocol. Knockdown efficiency was assessed 24-48 hours post-transfection.

#### Xenograft model for tumor growth delay

All animal work was done according to the ARRIVE guidelines (Kilkenny et al., 2010) under project licenses P4B738A3B and P8484EDAE. RT112 bladder cancer xenografts were established by implanting 100 μL (5 × 10^6^) cells with Matrigel (1:2 ratio of Matrigel and cold PBS with cells) subcutaneously into the flank of CD-1 nude mice. Mice were randomized to the following treatment groups using the “random” function on Excel: control (no treatment), 25 mg/kg CB-5083 (diluted in 5% DMSO, 0.5% methylcellulose and 1% Tween-80 in ddH_2_O) via intraperitoneal injection, radiotherapy only (6 Gy, single fraction) and radiotherapy combined with CB-5083, where the drug was administered 6 hours before radiotherapy. Treatments were initiated when tumor size reached 50 mm^3^. Tumor progression was monitored trice weekly with callipers, by the width, height and length x π/6. Kaplan-Meier survival analyses were used to measure the time to treble tumor volume in CD-1 nude mice, and statistical significance (p < 0.05) was determined using the Mantel-Cox test.

#### Pharmacokinetic analysis of CB-5083 concentration

Tumor cell xenografts were established in CD-1 nude mice as above. Treatment was given once tumors reached 50 mm^3^. Mice were treated at 25 mg/kg CB-5083 via intraperitoneal injection for 6 hours, and plasma, bladder, kidney, liver and tumor were collected to determine the concentration of CB-5083 (μM) by high performance liquid chromatography (HPLC) analysis.

#### Colony formation assay

Cells were seeded at appropriate densities in triplicate and irradiated with 0, 2, 4, 6 and 8 Gy. Cells were cultured for 8-10 days to allow colonies to form. Colonies were fixed and stained with 0.5% crystal violet in dH_2_O and 20% methanol for 5-10 minutes. Colonies were counted manually or by using GelCount colony counter (Oxford Optronix). The surviving fraction was determined by normalizing the number of colonies in each condition to the unirradiated control. For drug-only clonogenic assays, cells were normalized to DMSO control. All experiments were conducted in triplicate across technical replicates.

#### Immunofluorescence microscopy and irradiation

Cells cultured on 10 mm No. 1 cover glasses (VWR) were pre-sensitized in complete medium containing 10 μg/mL Hoechst 33342 20 minutes prior to laser micro-irradiation or ionising radiation. S phase cells were marked by treatment with 10 μM EdU for 20 minutes before DNA damage induction, after which medium containing drugs was replaced with fresh complete medium. For laser micro-irradiation, cells were irradiated using 355 nm pulsed laser connected to a Nikon TE2000 microscope. For ionising radiation, cells were irradiated at a dose rate of 1.5 Gy/min using Gamma-Service Medical GmbH GSR D1 irradiator. Cells were allowed to recover for indicated times prior to permeabilisation with 0.3% Triton X-100 for 10 minutes at room temperature (RT). Cells were fixed with ice cold 4% PFA in PBS for 15 minutes at RT, and samples were blocked by incubation in 5% BSA in PBS for 1 hour at RT or overnight at 4°C. Incubation with indicated primary and secondary antibodies was done in 2.5% BSA in PBS for 1 hour at RT, and coverslips were mounted on microscopy slides using mounting reagent with DAPI (Invitrogen). For laser micro-irradiation experiments and RPA, γH2AX, MRE11, 53BP1, REV7, RAD51 and RAD52 foci, soluble proteins were pre-extracted with 25 mM HEPES (pH 7.4), 50 mM NaCl, 1 mM EDTA, 3 mM MgCl_2_, 300 mM sucrose and 0.5% Triton X-100 for 3-5 minutes on ice. Fluorescent foci and laser-induced damage areas were imaged using a Nikon Ni-E epifluorescent microscope under a 60X objective (for Figures S2 and S4) or Zeiss 710 confocal microscopy using either a 40X or 63X objective (for all the rest of the figures). All microscopy images were analyzed with FIJI (ImageJ) software.

#### BrdU foci detection without denaturation

Cells cultured on 10 mm No. 1 cover glasses (VWR) were incubated with 10 μM BrdU for 24 hours to visualize single-stranded DNA. Cells were treated with 10 μM CB-5083 or 100 μM Mirin for the last 2 and 3 hours, respectively. Ten μM EdU was added 20 minutes prior to fixation to mark S phase. Cells were pre-extracted as above, fixed and the number of BrdU foci imaged using epifluorescence microscopy. For siRNA treatments, cells were treated with siRNA for 48 hours total using RNAiMAX as mentioned. BrdU was added to cells in the last 24 hours before harvesting.

#### Immunohistochemistry and H&E staining

Primary antibody for p97/VCP was diluted at 1:2,000 in 3% BSA and incubated overnight at 4°C, followed by biotinylated secondary antibody (Vector) for 15 minutes at RT. Slides were counterstained with hematoxylin. Immunostaining was scanned using 40X magnification with the Aperio ScanScope CS2 scanner and images were captured using Leica Aperio Image Scope software. Tissue areas were outlined by a consultant histopathologist (LB), John Radcliffe Hospital, Oxford. Staining intensity was determined independently by two observers (SK+AK): 0 (negative), 1+ (weak), 2+ (moderate), 3+ (strong). Both scorers used reference images with typical scoring values to determine the intensity of each area, and an overall consensus score was reached.

#### Cellular fractionation

Cells were fractionated as described previously (Méndez and Stillman, 2000). In brief, the cytoplasmic fraction was isolated by suspending cells in buffer A (10 mM HEPES, 10 mM KCl, 1.5 mM MgCl_2_ 2, 0.34 M sucrose, 10% glycerol) freshly supplemented with protease and phosphatase inhibitors, NEM and 0.1% Triton X-100. Nuclei were ruptured to release the nucleoplasm using hypotonic buffer B (10 mM HEPES, 3 mM EDTA, 0.2 mM EGTA, pH 8.0). The chromatin was washed with benzonase buffer (25 mM Tris-HCl, pH 8.0, 20 mM NaCl, 2 mM MgCl_2_) and digested with benzonase (500 U/ml) while rotating at 10 rpm for 30 min at room temperature. Protein concentrations were measured using the RC DC Protein Assay kit (BioRad).

#### Immunoblot

Cell lysates were denatured by boiling in Laemmli sample buffer, separated by SDS-PAGE and transferred (Bio-Rad Trans-Blot Turbo system or wet transfer) to 0.2-μm nitrocellulose membrane. Membranes blocked with Odyssey blocking buffer (Li-Cor Biosciences) or 5% milk for 1 hour at room temperature. Membranes were incubated overnight with primary antibodies (Li-Cor antibody diluent or 5% BSA in TBS-T, pH 7.4). Membranes were washed three times with PBS-T (0.1% Tween-20) and incubated with secondary antibodies (IRDye800 or 700CW secondary antibodies or secondary antibodies coupled to HRP) for 1 hour at room temperature. After three additional washes with PBS-T, membranes were detected using a LI-COR Odyssey Fc (LI-COR Biosciences) or ECL-based chemiluminescence with Bio-Rad gel imaging system.

#### Protein co-immunoprecipitation (Co-IP)

Cells were transfected with the plasmids of interest using FuGENE® HD transfection reagent (Promega; #E2311) and allowed to express for 20 hours. In case of doxycycline (DOX)-induced expression of Strep-p97-EQ, cells were treated with DOX (1μg/ml) for 18 hours to induce protein expression. Cells were then treated with DMSO/CB-5083 (10 μM for 4 hours) and/or IR (10 Gy with 2-3 hours of recovery). Cells were harvested by trypsinisation and rinsed twice with ice-cold PBS. Chromatin fraction was isolated as described above and digested with benzonase and RNase at 4°C, 15 rpm for 2 hours. Digested chromatin was centrifuged at 10,000 x g, at 4°C for 10 minutes and clear supernatant was transferred into a new tube. Supernatant was diluted 2-3 times with IP buffer (50 mM Tris-HCl pH 7.4; 150 mM NaCl; 1 mM EDTA; 0.5% Triton X-100; 20mM NEM; protease and phosphatase inhibitors) and incubated with pre-washed anti-HA magnetic beads (Thermo Fisher; #88836) or MagStrep “type3” XT beads (IBA LifeSciences; #2-4090-002). IP was performed according to the manufacturer’s protocol. Proteins were eluted in Laemmli sample buffer (2X) and analyzed by Western Blotting.

In case of endogenous p97 Co-IP, HEK293 cells were treated as indicated and fractionated to isolate chromatin. The chromatin fraction was digested as above and diluted 2 times with IP buffer (50 mM Tris-HCl pH 7.4; 150 mM NaCl; 1 mM EDTA; 0.1% Triton X-100; 2 mM MgCl_2_; 20 mM NEM, protease and phosphatase inhibitors). The diluted sample was used as input and incubated at 4°C, 15 rpm first with p97 (Proteintech; #10736-1-AP) or control IgG (non-commercial/KR Lab) antibodies for 1-2 hours and then with protein A magnetic beads (NEB; #S1425S) for another 2 hours. IP was performed according to the manufacturer’s protocol. Proteins were eluted in Laemmli sample buffer (2X) and analyzed by Western Blotting.

#### MRE11 pull-down for mass spectrometry analysis

For mass spectrometry of MRE11 interactome, T24 cells were plated on 15 cm plates and transfected with 20 μg pCDNA3.3 vector containing HA-MRE11 per plate, with FuGene transfection reagent in a 1:3 ratio. Cells were treated with 5 Gy IR in a cesium-137 Gamma-Service Medical GmbH GSR D1 irradiator and recovered for 4 hours. Cells were harvested and lysed in 50 mM HEPES pH 7.5, 100 mM NaCl, 10 mM EDTA, 1% w/v Triton X-100, 4 mM sodium pyrophoshate, 2mM sodium orthovanadate, 10 mM sodium fluoride, 50 mM β-glycerophosphate and Roche Complete Mini Protease inhibitor cocktail. Cell lysate was incubated with a mixture of 25 μL protein A and 25 uL protein G dynabeads which were pre-washed with lysis buffer for 1 hour to pre-clear sample. HA-conjugated magnetic beads (Pierce) were also washed in lysis buffer, and sample incubated with these beads at 4°C for 1 hour with rotation. Beads were then washed six times with lysis buffer as above but containing 0.5 M NaCl instead of 100 mM NaCl. Elution was carried out with HA peptide (2 mg/mL), 20-100 μL for 2 hours at room temperate. After elution, protein was precipitated using chloroform/methanol, then trypsin digestion was carried out (Fischer et al., 2012).

#### Stable Isotope Labeling with Amino Acids in Cell Culture (SILAC)/mass spectrometry (MS)

For quantitative Mass-Spectrometry, Flip-In T-Rex HEK293T/p97-E578Q-Strep cell lines were first SILAC labeled. After induction of p97-E578Q-Strep with doxycycline (DOX) and treatment with 10 Gy of IR, nuclear/chromatin fractions were extracted, treated with benzonase, and the p97 interactors in this fraction were pulled down with Strep-TACTIN beads (IBA).

#### Sample preparation and analysis by mass spectrometry

Pulled down proteins were reduced, alkylated and then precipitated using chloroform/methanol protein precipitation (Fischer et al., 2012). Protein precipitates were solubilised in 6 M urea in 100 mM Tris pH 7.4, digested with trypsin (overnight at 37°C; Promega), de-salted using C18 Sep Pak column cartridges (Waters, Milford, MA, USA) and dried using a speed vacuum. Peptides were resuspended in water with 0.1% trifluoroacetic acid (TFA) and 2% acetonitrile.

MRE11 pulled down tryptic peptides were analyzed by nano-ultra performance liquid chromatography tandem mass spectrometry (UPLC-MS/MS) using a Waters nanoAcquitiy UPLC system connected to a LTQ Orbitrap Velos mass spectrometer (Thermo), as described previously (Fischer et al., 2012). In brief, tryptic peptides were separated on a C18 reverse-phase column (Waters, nAcquity, 75μm x 25cm, 1.7μm particle size) using a multistep gradient from 1 to 40% acetonitrile in 60 min at a flow rate of 250 nl/min. Full MS scans were acquired over the m/z range of 300 – 1500 at 60000 resolution. MS/MS data was acquired in a data dependent manner by selecting the top 20 most abundant precursor ions for CID fragmentation (Collision energy of 35). Proteins were identified in MASCOT (v2.3), using the Human UniProt_SwissProt database (retrieved 20150204; containing 20,274 sequences). MASCOT data results were filtered by applying a significance threshold at p < 0.001. The mass spectrometry proteomics data have been deposited to the ProteomeXchange Consortium via the PRIDE partner repository with the dataset identifier PXD016279 (Perez-Riverol et al., 2019).

p97 pulled down tryptic SILAC labeled peptides were analyzed by nano-UPLC-MS/MS using a Dionex Ultimate 3000 nano-UPLC coupled to a Q-Exactive mass spectrometer (Thermo Fisher Scientific), as described previously (Vaz et al., 2016). Peptides were loaded onto a trap column (PepMapC18; 300μm x 5mm, 5μm particle size, Thermo Fischer) for 1 min at a flow rate of 20 μl/min and separated on an EASY-spray column (75 μm X 500 mm, 2 μm particle size; Thermo Scientific) with a linear gradient from 2% to 35% acetonitrile in 0.1% formic acid and 5% DMSO with a 250 nL/min flow rate in 60 min. Mass spectra were acquired in a data-dependent top15 method with the automatic switch between full MS-scan and MS/MS. Full MS scans were acquired in the Orbitrap with m/z range of 380–1800 and at a resolution of 70K (AGC target at 3e6 ions, maximum injection time of 100 ms). Precursor ions (charge state > = 2) were sequentially isolated in the Quad (m/z 1.6 window) and fragmented on the HCD cell (normalized collision energy of 28%). MS/MS data was obtained in the Orbitrap at a resolution of 17500 with a maximum acquisition time of 128ms, an AGC target of 1e5 and a dynamic exclusion of 27 s. SILAC MS/MS data was analyzed using Proteome Discoverer (v1.4.0.288; ThermoFisher). Peptide and protein identification was performed against the human UniProt-SwissProt database (retrieved 20150204; containing 20,274 sequences) using MASCOT (sequences) and subsequently SILAC ratios were determined using the Proteome Discoverer software algorithm recommended settings. Results for RAD50 protein were selected for this study.

#### High performance liquid chromatography (HPLC) analysis

CB-5083 calibration standards were prepared in control mouse plasma. Tissue samples were defrosted, weighed and homogenized in deionised water giving a five-fold dilution. Plasma samples were used without dilution. Aliquots of each sample or standard was taken and Gefitinib added as internal standard prior to extraction using ethyl acetate. Samples were dried down and reconstituted in mobile phase for analysis.

Separation was performed on a Waters Alliance e2695 with a Waters X-Bridge C-18 3.5 μm 2.1 × 50 mm column. Analytes were detected using a Waters Acquity QDa mass detector monitoring m/z 414.5 for CB 5083 and m/z 447.2 for Gefitinib in positive ion mode. Mobile phase A was 10 mM ammonium formate pH 9.7, mobile phase B was acetonitrile. A gradient at 0.4 mL/min of 35%–90% B was run over 2 minutes with a total run time of 6 minutes.

#### Reporter assays

To measure homologous recombination (HR) efficiency, 5x10^5^ U2OS DR-GFP cells were seeded in 6-well plates. Cells were treated with indicated siRNAs and transfected the following day with 4 μg of I-SceI plasmid (gift from Dr. Timothy Humphrey, University of Oxford) using Lipofectamine 3000 as per manufacturer’s instructions (Invitrogen). For CB-5083 treatment, cells were treated for 6 hours at 10 μM prior to I-SceI transfection. Cells were recovered in fresh complete media for at least 24 hours and GFP-positive cells were determined using BD FACS DIVA software. The data were then normalized to I-SceI positive control and analyzed using FlowJo V10. Statistical significance was determined using one-way ANOVA. To measure single-strand annealing (SSA) efficiency, 5x10^5^ U2OS SA-GFP reporter cells were seeded in 6-well plates. Cells were transfected, treated and analyzed exactly as for HR assay, however siRAD52 was used as a positive control instead of siRAD51.

### Quantification and statistical analysis

All statistical analyses were performed using GraphPad Prism 8 software unless otherwise specified. The type of statistical test used is indicated in figure legends. All data are representative of 3 independent experiments unless otherwise stated. Sample sizes are included in the figure legends. A p value of p ≤ 0.05 was considered statistically significant.

## Supplemental information

Document S1. Figures S1–S7

Table S1. Mass spectrometry analysis of HA-MRE11 pull-down from T24 cells, related to Figure 1 MRE11 pull down in T24 cells and subsequent mass-spectrometry identification of its interactome. The data analysis has been described in STAR Methods in detail.

Document S2. Article plus supplemental information
