# Supplementary material for: p97/VCP inhibition causes excessive MRE11-dependent DNA end resection promoting cell killing after ionizing radiation
Source: Cell Rep. 2021 May 25;35(8):109153. doi: 10.1016/j.celrep.2021.109153 (PMC8170441; doi:10.1016/j.celrep.2021.109153)
Supplement: Document S1. Figures S1–S7 [file mmc1.pdf]

Cell Reports, Volume *35*

Supplemental information

p97/VCP inhibition causes excessive MRE11-dependent DNA end resection promoting cell killing after ionizing radiation

Susan Kilgas, Abhay Narayan Singh, Salome Paillas, Chee-Kin Then, Ignacio Torrecilla, Judith Nicholson, Lisa Browning, Iolanda Vendrell, Rebecca Konietzny, Benedikt M. Kessler, Anne E. Kiltie, and Kristijan Ramadan

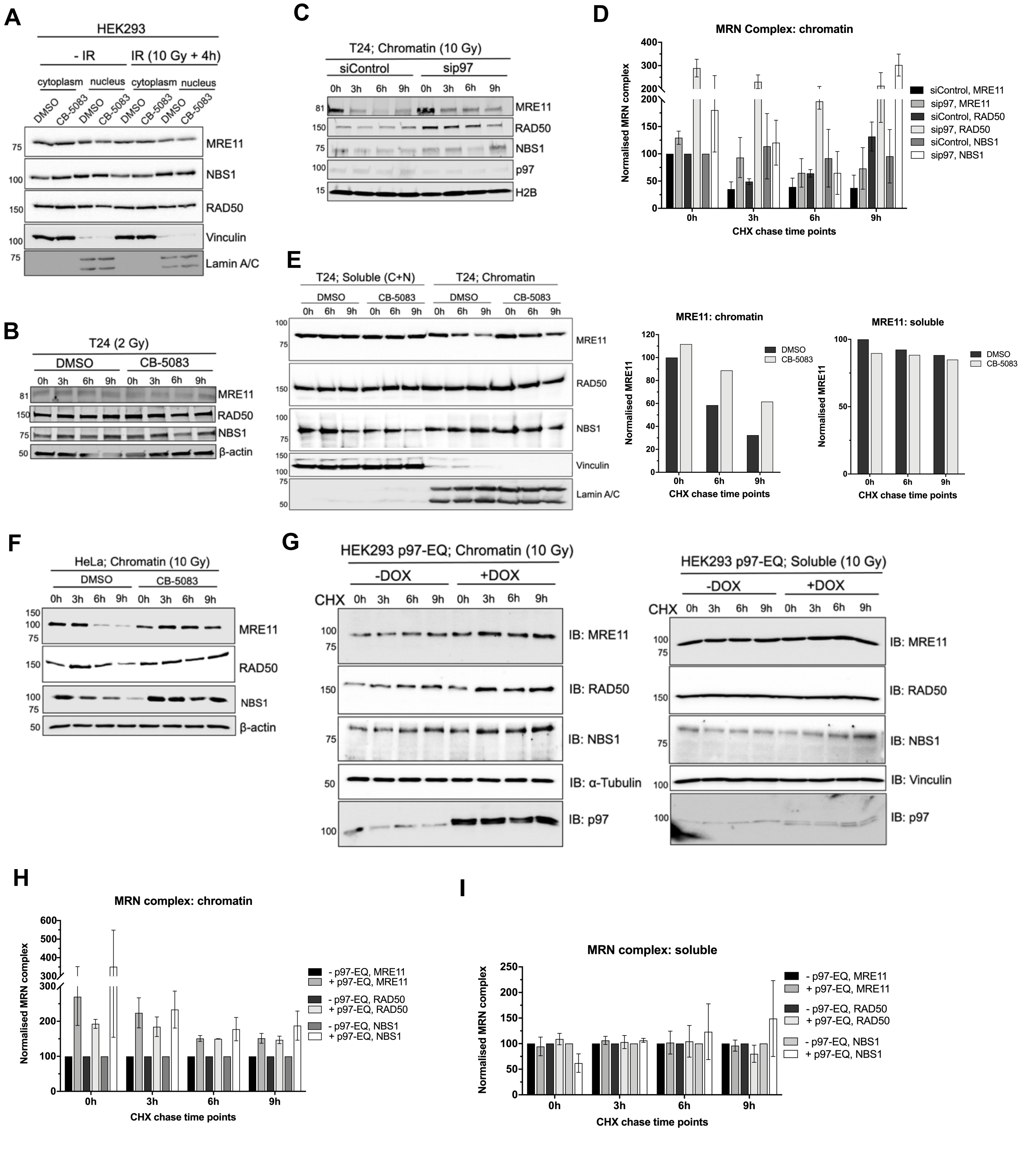

Supplementary Figure 1 (Related to Figure 1): p97 regulates MRN complex retention on chromatin after IR. A) Total levels of MRN (+/-10 Gy IR; 4 h recovery) in CB-5083-treated HEK293 cells; n=2. **B**) Total levels of MRN (+/- 2 Gy IR) in CB-5083-treated T24 cells. **C**) T24 cells treated with CHX with or without p97 siRNA upon 10 Gy IR. **D**) Quantifications of MRN levels on chromatin. Data is normalised to H2B loading control. Data are represented as mean +/- SEM; n=2. **E)** T24 cells treated with CHX with or without CB-5083 upon 10 Gy IR. Soluble and insoluble fractions shown on the same gel. Quantifications shown on the right. F) HeLa cells treated with CHX with or without CB-5083 upon 10 Gy IR; n=2. **G**) Doxycycline (DOX)-inducible HEK293 Flip-In p97-EQ cells treated with CHX and 10 Gy IR. **H)** Quantifications of MRN levels on chromatin and in soluble (**I**) fraction; Data are represented as mean +/- SEM; n=2.

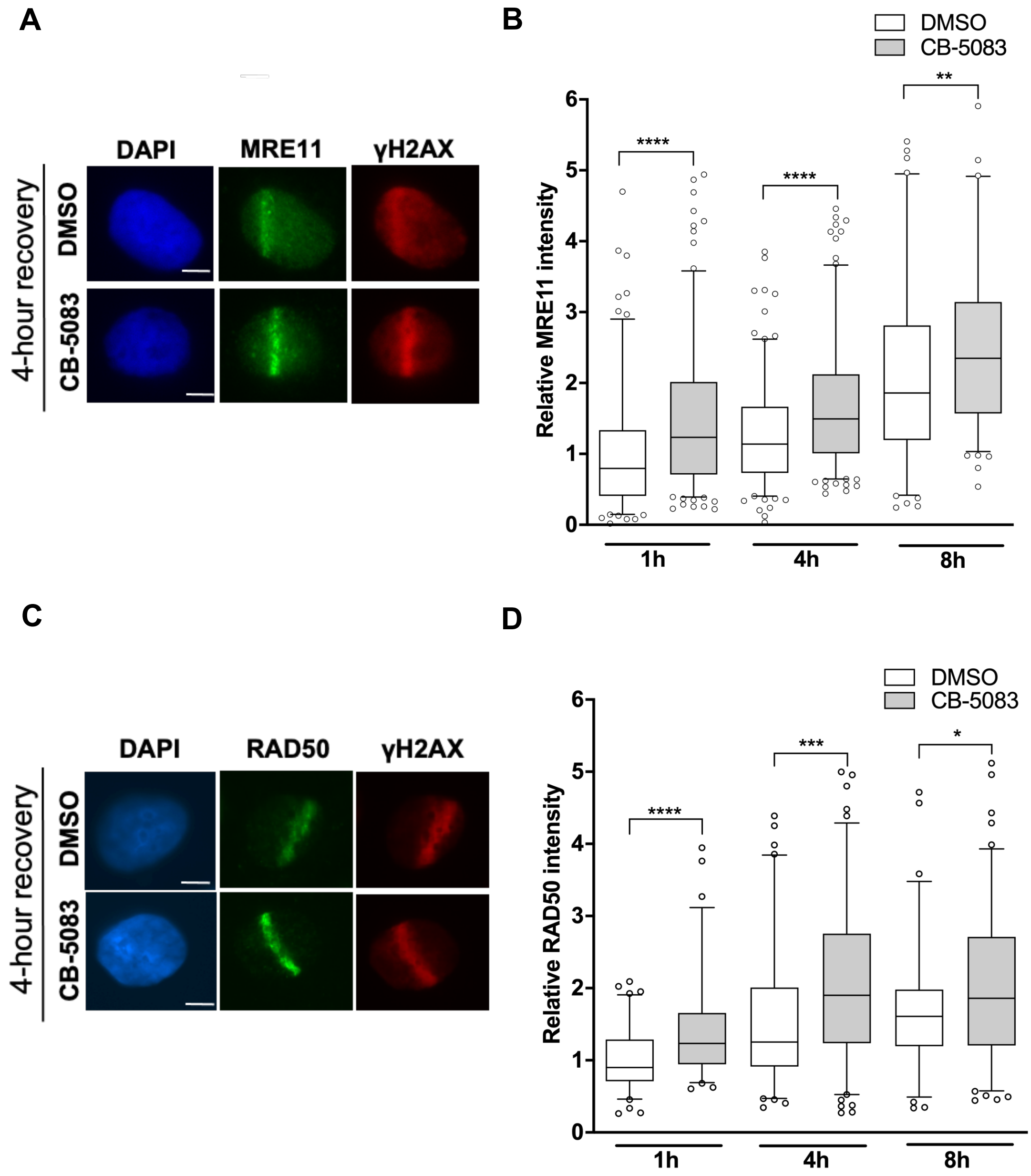

Supplementary Figure 2 (Related to Figure 1): Inhibition of p97 causes MRE11 and RAD50 accumulation on UV-A-laser-induced DNA damage sites. A) Representative images of MRE11 recruitment to DNA damage in T24 cells pre-sensitised with Hoechst (10 µg/mL) (Scale bar: 5μm). **B)** Quantifications of MRE11 intensity at UV-laser stripes. **C)** Representative images of RAD50 recruitment to DNA damage (as in A) (Scale bar: 5μm). **D)** Quantifications of RAD50 intensity at UV-laser stripes. MRE11 and RAD50 intensity was quantified on DNA damage and normalised to the intensity at the earliest time point in DMSO control. For both graphs, data is presented as box plot, and values beyond the 5th and 95th percentiles are shown as individual data points; n=3 for 1-hour and 4-hour recovery; n=2 for 8-hour recovery; Mann-Whitney test. * $p<0.05$; ** $p<0.01$; **** $p<0.0001$.

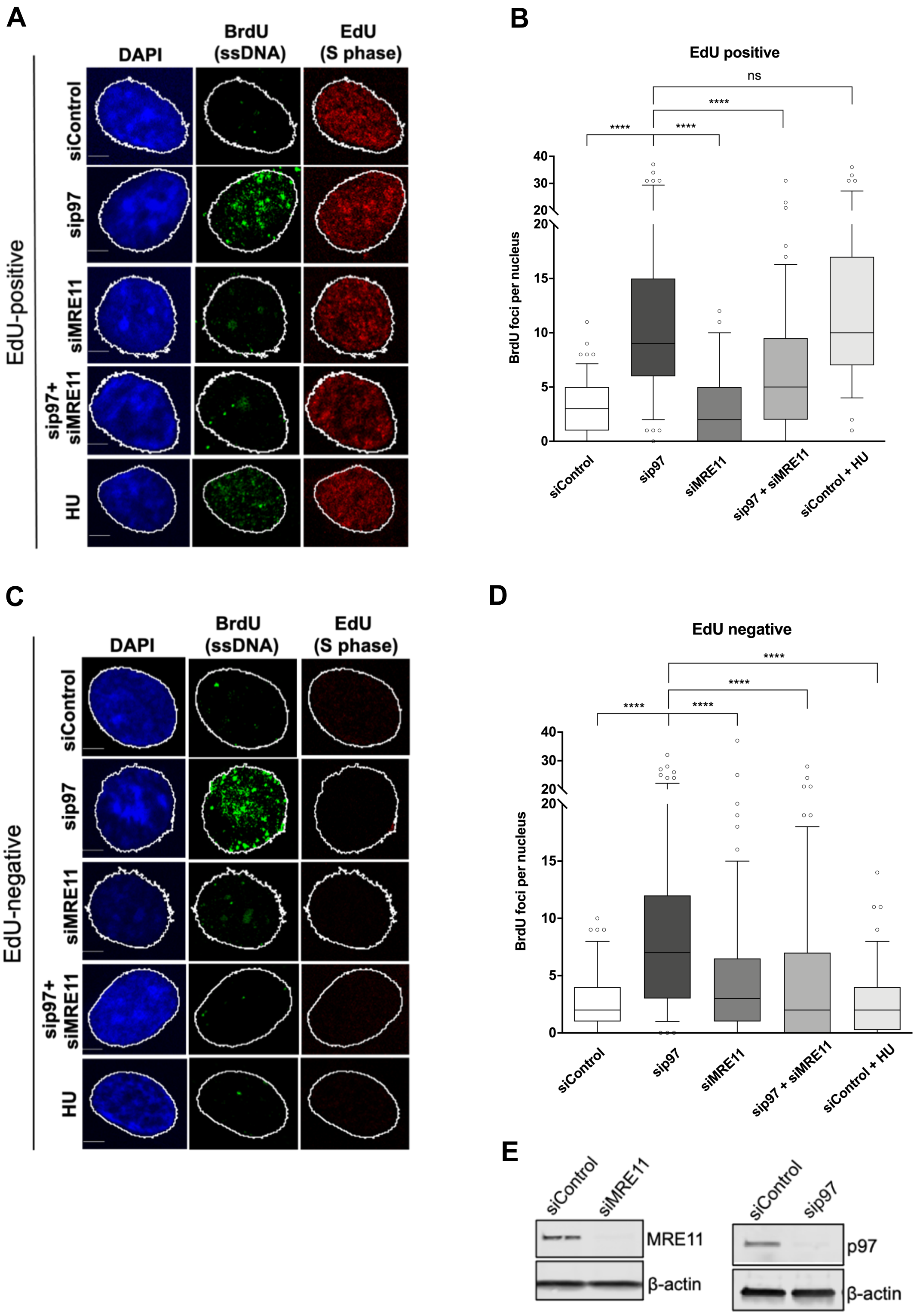

Supplementary Figure 3 (Related to Figure 2). Depletion of p97 causes excessive ssDNA generation mediated by MRE11. Representative images of BrdU foci in EdU-positive (**A**) and –negative (**C**) T24 cells after siRNA-mediated knockdown of p97, MRE11, or both. Hydroxyurea (HU) serves as a positive control in EdU positive cells (Scale bar: 5μm). (**B**) and (**D**): Quantifications of (A) and (C). For both graphs, data is presented as box plot, and values beyond the 5th and 95th percentiles are shown as individual data points; n=2; Kruskal-Wallis test with Dunn's multiple comparisons. NS, not significant; ****p<0.0001; **(E)** Western Blots of p97 and MRE11 knockdown.

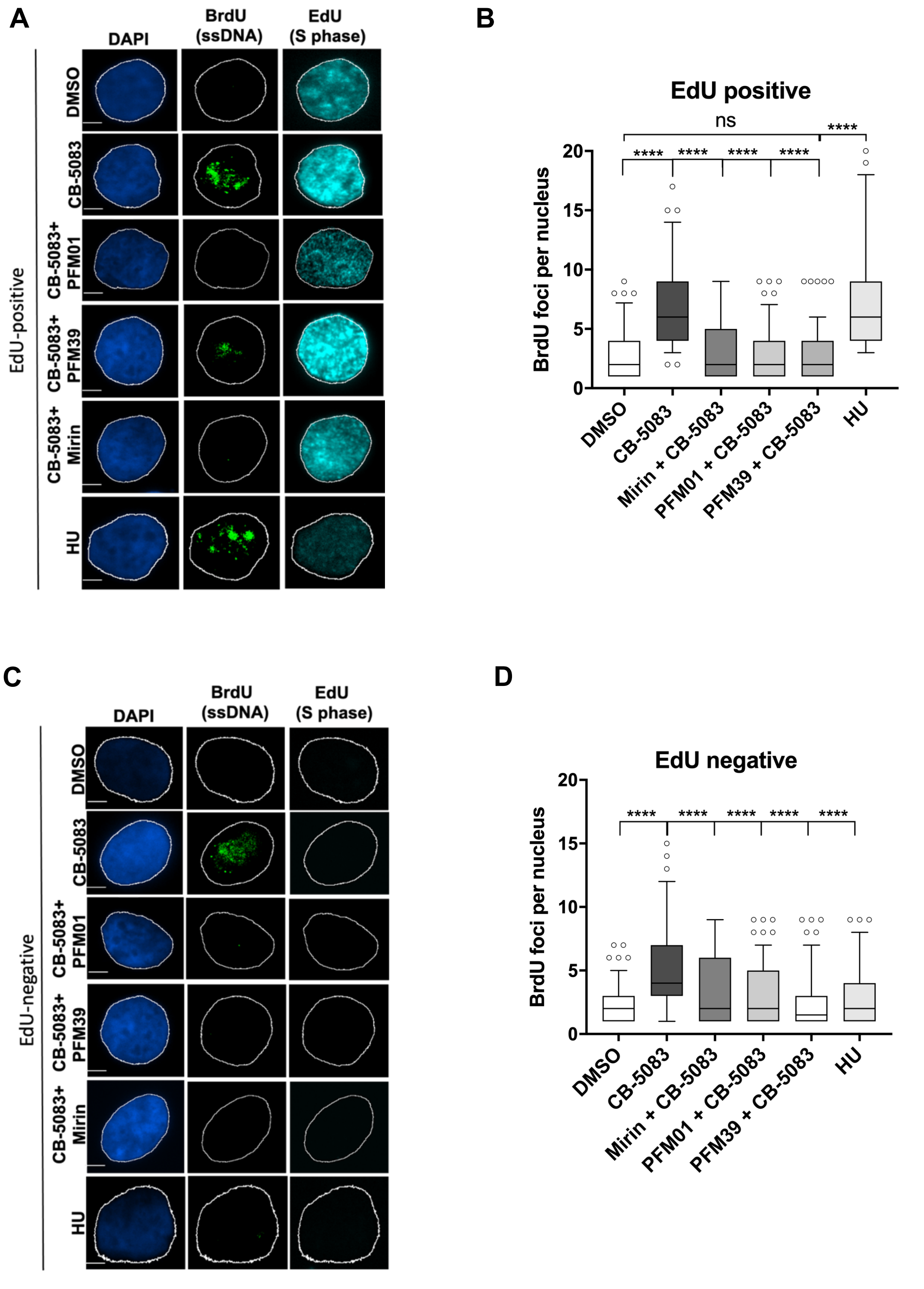

Supplementary Figure 4 (Related to Figure 2). MRE11 endo- and exonuclease inhibitors rescue CB-5083-induced excessive ssDNA generation. Representative images of BrdU foci in EdU-positive (**A**) and -negative (**C**) T24 cells after treatment with CB-5083, or in combination with Mirin, PFM01 or PFM39 (Scale bar: 5μm). **B**) and **D**) Quantifications of (A) and (C), respectively. Hydroxyurea (HU) serves as a positive control in EdU positive cells. For both graphs, data is presented as box plot, and values beyond the 5th and 95th percentiles are shown as individual data points; n=2; Kruskal-Wallis test with Dunn's multiple comparisons. NS, not significant; ****p<0.0001.

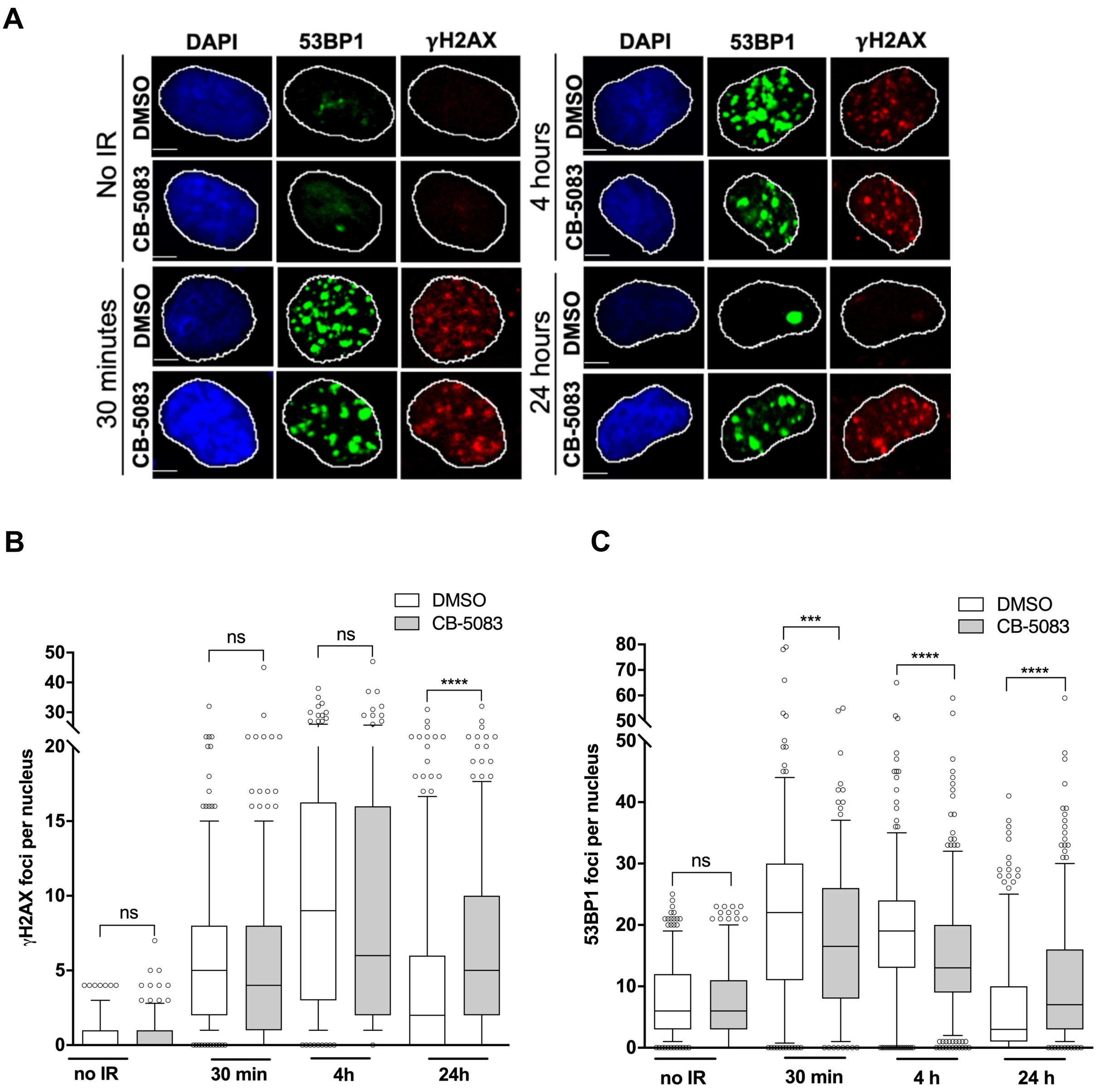

Supplementary Figure 5 (Related to Figure 4): Inhibiting p97 by CB-5083 causes early 53BP1 recruitment defect and DNA repair delay upon IR. A) Representative 53BP1 and γH2AX foci in p97-inhibited (CB-5083) T24 cells after 2 Gy IR (Scale bar: 5μm). **B)** 53BP1 and (**C**) γH2AX foci quantifications in CB-5083-treated T24 cells. For both graphs, data is presented as box plot, and values beyond the 5th and 95th percentiles are shown as individual data points; n=3; Mann-Whitney test. NS, not significant; *** p<0.001; **** p<0.0001.

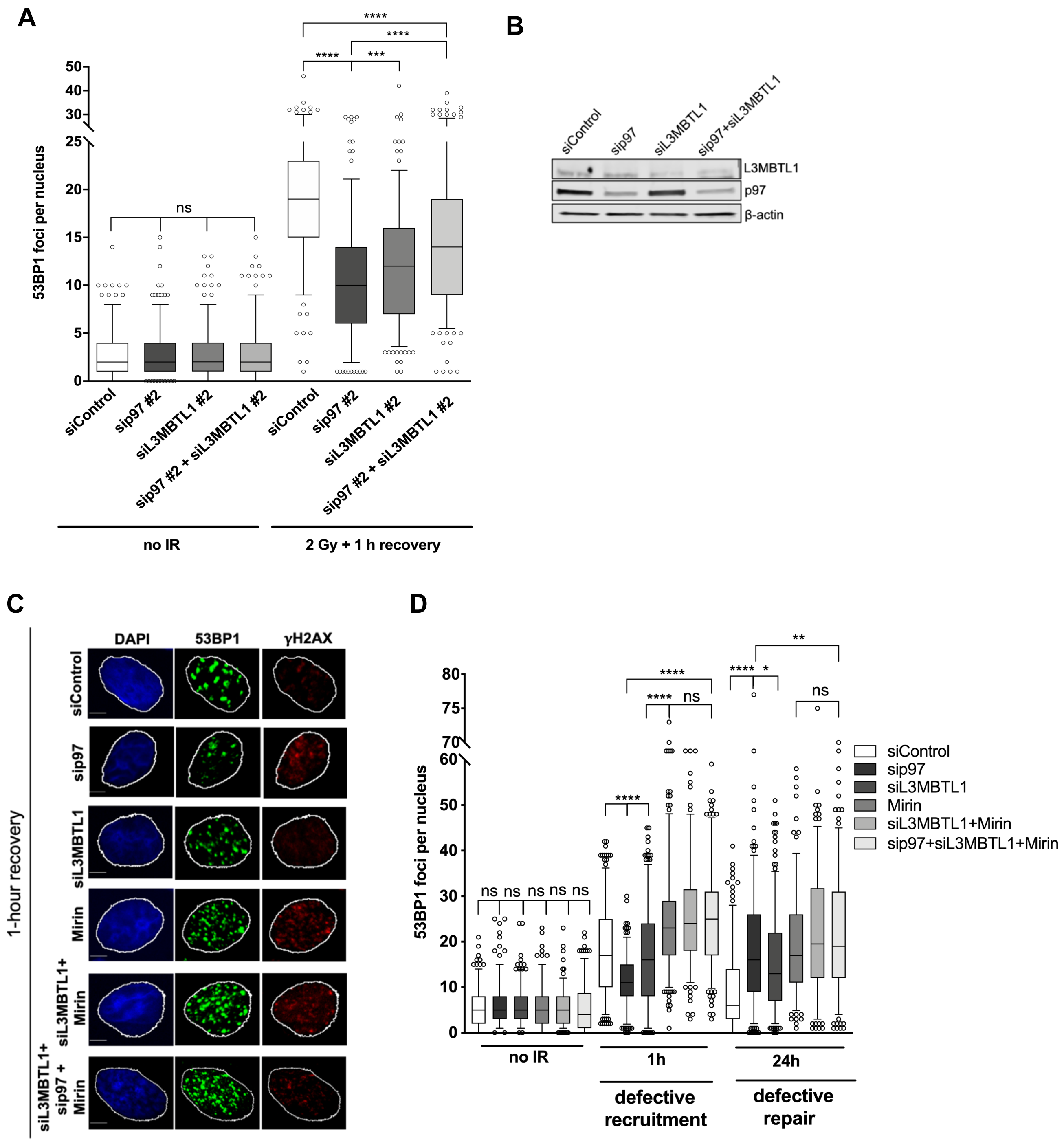

Supplementary Figure 6 (Related to Figure 5): p97-mediated recruitment of 53BP1 is only partially dependent on L3MBTL1. A) Quantifications of 53BP1 foci in p97- and L3MBTL1-depleted T24 cells upon 2 Gy IR; n=2; Kruskal-Wallis test with Dunn's multiple comparisons; NS, not significant; **** p<0.0001. **B)** Western Blot of p97 and L3MBTL1 knockdown. **C)** Representative images of 53BP1 foci in T24 cells after indicated siRNA/drug treatments and recovery from 2 Gy IR (Scale bar: 5μm). **D)** Quantifications of (C). Values for sip97 are taken from Figure 5K but presented separately in two graphs for clarity of presentation; n=3; Kruskal-Wallis test with Dunn's multiple comparisons. NS, not significant; *** p<0.001; **** p<0.0001. For all graphs, data is presented as box plot, and values beyond the 5th and 95th percentiles are shown as individual data points.

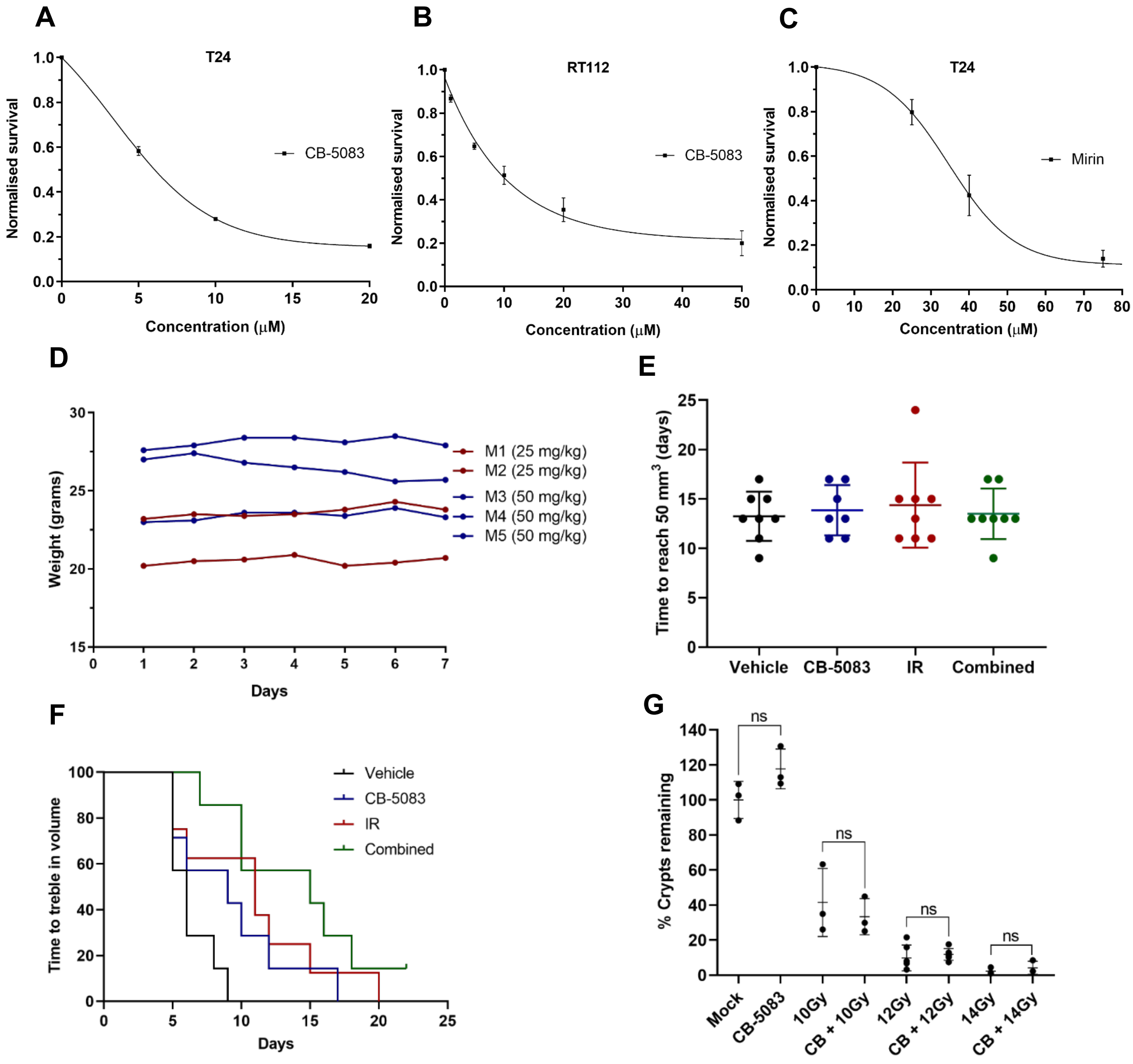

Supplementary Figure 7 (Related to Figure 7): Effects of CB-5083 and Mirin on cell survival and *in vivo* effects of CB-5083 in combination with IR. Survival of T24 (**A**) and RT112 (**B**) cells upon 6-hour treatment with CB-5083. IC10 (T24: 1 µM; RT112: 1 µM) and IC50 (T24: 6 µM; RT112: 10 µM). Data is normalised to DMSO control; n=3; data are represented as mean +/- SEM. **C**) Survival of T24 cells upon 24-hour treatment with the MRE11 inhibitor Mirin; IC10 (≈18 µM) and IC50 (37 µM). Data is normalised to DMSO control; n=3; data are represented as mean +/- SEM. **D**) Weight changes in 6-7-week-old CD-1 nude mice after a single intraperitoneal injection of 25 mg/kg (n=2 mice) or 50 mg/kg of CB-5083 (n=3 mice); "M" stands for "mouse". **E**) Time for CD-1 nude RT112 xenografts to reach 50 mm^3 in tumour size. **F**) Time to treble in tumour volume presented as Kaplan-Meier survival curve (n=8 mice per group). **G)** Percent of remaining crypts after 10 Gy (n=3 mice), 12 Gy (n=6 mice) and 14 Gy (n=3 mice) IR, or in combination with 25 mg/kg CB-5083 (6-hour treatment); unpaired t-test. NS, not significant.
